# DCT-Based Transparent Object Segmentation Network with Cooperative CNN–Transformer Branches

**DOI:** 10.3390/s26216935

**Published:** 2026-11-02

**Authors:** Jianfeng Liu, Hedong Liu, Haofeng Hu

**Affiliations:** 1 School of Marine Science and Technology, Tianjin University, Tianjin 300072, China; liujf_007@tju.edu.cn; 2 China-Belarus Belt and Road Joint Laboratory on Intelligent Perception in Extreme Environments, Tianjin 300072, China; 3 School of Precision Instrument and Opto-Electronics Engineering, Tianjin University, Tianjin 300072, China

**Keywords:** transparent object segmentation, image sensors, dual-branch neural network, frequency feature decoupling, cross-branch feature interaction, adaptive attention feature fusion

## Abstract

Transparent object segmentation is a critical computer vision task for industrial and mobile robotic perception, which is widely deployed in intelligent cleaning robots and unmanned aerial vehicles (UAVs) equipped with visual image sensors. However, the perception performance of mainstream image sensors is severely hindered by the unique optical characteristics of transparent objects, including light transmission, complex reflective noise and indistinct object textures, resulting in inaccurate segmentation results in actual robot sensing scenarios. Precise extraction of transparent object boundaries and suppression of background semantic interference are essential for high-quality segmentation. Existing transparent object segmentation methods either focus on semantic or boundary information independently, or they extract boundary features merely from shallow layers during joint modeling, which always causes incomplete boundary feature extraction. Considering that transparent object boundaries correspond to high-frequency features while low-frequency components carry dominant semantic information, we develop a DCT-based segmentation network with cooperative CNN–Transformer branches. DCT decomposes input images into high- and low-frequency components, where CNN and Transformer branches learn frequency-specific features separately. We embed an interactive information module (IIM) in dual encoders to realize bidirectional cross-frequency feature interaction, strengthen weak boundaries, eliminate incomplete target regions caused by reflection noise, and reduce false edges. In decoders, the hybrid adaptive calibration module (HACM) fuses dual-branch semantic and boundary features, alleviates feature distribution discrepancies, and optimizes object positional information. A cascaded head further achieves effective coarse-to-fine multi-scale feature aggregation to adapt to multi-distance sensing scenarios for robots. Our experimental results on five public datasets verify that our method outperforms current mainstream state-of-the-art segmentation methods, effectively compensating for the limitation of traditional image sensors in transparent object recognition and offering a promising development in the environmental perception robustness of cleaning robots and UAVs.

## 1. Introduction

Transparent objects widely exist in both industrial production environments and daily robot working scenarios, including indoor scenarios for intelligent cleaning robots and low-altitude flight scenarios for unmanned aerial vehicles (UAVs). Most mobile and industrial robots rely on onboard image sensors as the core visual perception device to acquire environmental images. However, commercial image sensors exhibit inherent limitations when perceiving transparent targets. Transparent objects have distinctive optical properties of light transmission and specular reflection. During robot imaging, these characteristics easily cause texture confusion with complex backgrounds and produce faint, blurred object boundaries. Such imaging defects pose persistent challenges for practical computer vision and robotic perception applications [1,2,3]. Such representation defects severely limit the practical deployment of neural networks for various robotic vision tasks, including industrial automated sorting [4], industrial robotic visual perception [5], surface defect inspection [6], as well as civilian robot obstacle avoidance and environmental understanding for cleaning robots and UAVs [7].

**Figure 1:**
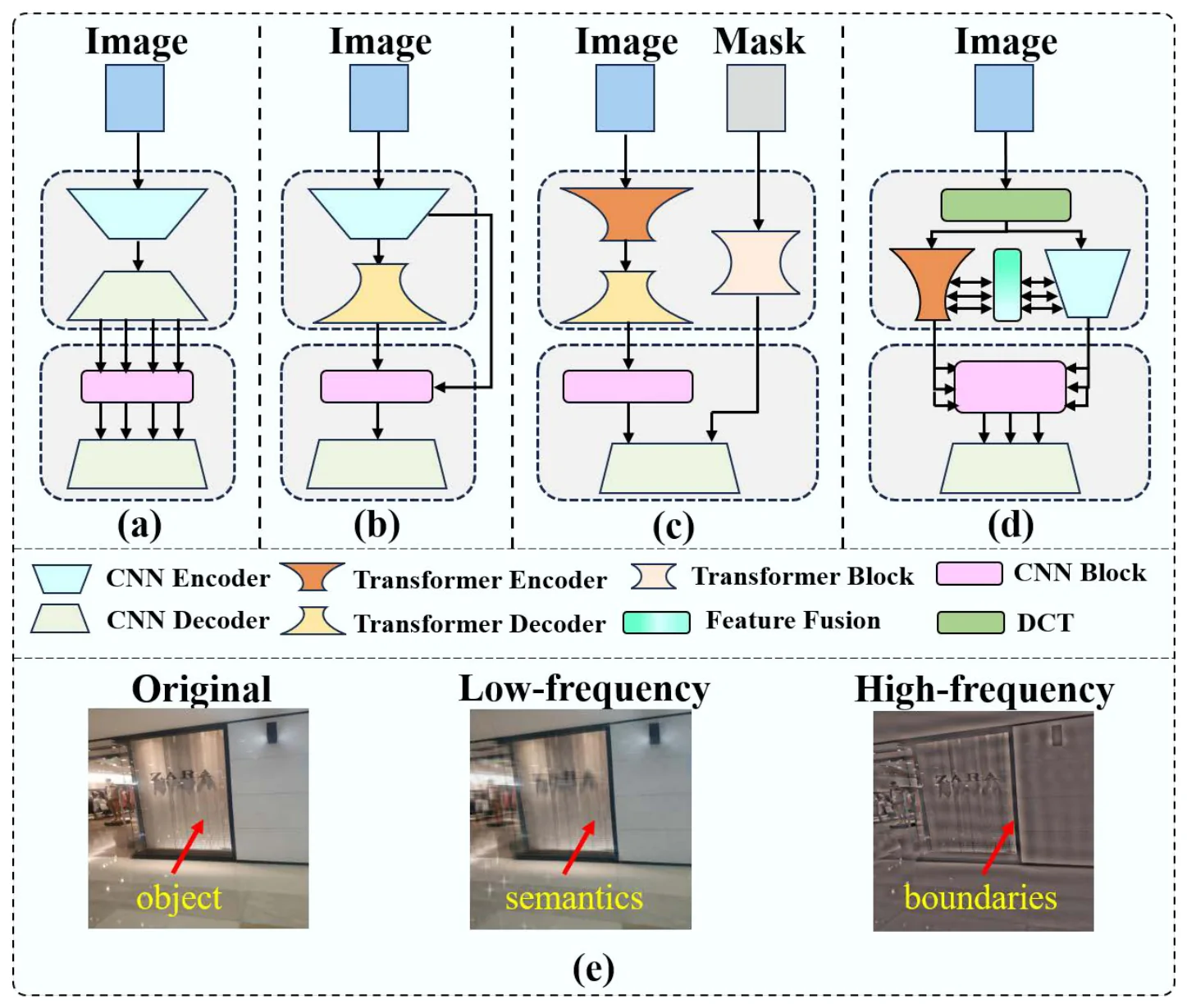
Four mainstream neural learning architectures for transparent object segmentation: (**a**) CNN-based architecture. (**b**) Mixed CNN–Transformer single-branch architecture. (**c**) Conventional dual-branch network. (**d**) Our proposed frequency-decoupled collaborative CNN–Transformer dual-branch learning architecture. (**e**) Our DCT transformation visualization of frequency features for transparent objects.

A straightforward strategy to segment transparent objects is to locate targets via edge detection. Nevertheless, reflective interference yields incomplete and blurry object boundaries, which leads to weakened feature discrimination [8]. Another category of context-driven methods suffers severe performance degradation, as textures of transparent targets heavily overlap with background regions and impair discriminative representation learning. Existing learning-based methods mostly adopt a single CNN or Transformer-based backbone to independently encode contextual semantics or boundary cues. As illustrated in Figure 1a–c, CNN-based networks are limited by localized convolution receptive fields and lack sufficient long-range dependency modeling capacity, which undermines global semantic representation. Transformer models excel at global modeling and possess inherent advantages in extracting the holistic information of transparent objects, yet they gradually degrade high-frequency information as network depth increases, resulting in inferior detail representation and degraded segmentation performance. It is noteworthy that existing dual-branch schemes merely concatenate CNN and Transformer outputs without dedicated cross-branch learning mechanisms. Their feature fusion is limited to the decoder stage, resulting in insufficient mutual guidance and complementary interaction between branches. As shown in Figure 1e, transparent object segmentation requires capturing low-frequency information such as surrounding background, textures, and colors. Meanwhile, high-frequency information including shape contours often provides pronounced responses at object boundaries, which can serve as a useful cue in distinguishing real scenes from virtual content formed by transmission and reflection of transparent objects. Nevertheless, both types of information suffer from inherent challenges caused by the reflection and light transmission properties of transparent objects. Specifically, edges tend to be blurred, and object semantics are easily confused with background information. Accordingly, relying on a single network paradigm may be insufficient to jointly capture both fine-grained boundaries and global semantics. It is necessary to construct a network that collaboratively processes high-frequency and low-frequency information for transparent object segmentation.

Based on the above analysis, we propose a collaborative CNN–Transformer network architecture to adequately process the high-frequency and low-frequency information of transparent objects, as illustrated in Figure 1d. Firstly, we employ DCT to separate the original RGB images in the frequency domain. The obtained low-frequency and high-frequency information is fed into the Transformer encoder and CNN encoder respectively for feature extraction. Then, to realize mutual feature guidance between the two branches and comprehensively extract semantic and boundary information during encoding, we embed the proposed interactive information module (IIM) within the dual-branch encoders. This module emphasizes areas with weak feature responses, particularly pixels near object boundaries. In a similar way, boundary features also receive selective enhancement under the guidance of the semantic branch, which reduces disturbances brought about by edge noise originating from backgrounds or inner areas of transparent objects. Furthermore, we propose a hybrid adaptive calibration module (HACM) within the decoder to alleviate inherent feature distribution mismatches between separated frequency branches. Unlike previous hybrid networks that simply adopt element-wise addition or concatenation, HACM dynamically reweights features via learnable weights to jointly refine discriminative global contextual and local boundary features. Following multi-scale representation learning paradigms, cascaded progressive aggregation layers are integrated to refine hierarchical multi-scale feature interactions. In summary, the core contributions of this work are threefold:

- Based on frequency transformation implemented by DCT, we have designed a collaborative CNN–Transformer dual-branch network to decompose transparent object features into low-frequency and high-frequency components and obtain richer multi-frequency features.
- We embed the interactive information module (IIM) in dual-branch encoders to strengthen weak boundary features and suppress transparent objects and background noise. After encoding, we design the hybrid adaptive calibration module (HACM) within the decoder to adaptively fuse multi-frequency features and optimize ambiguous pixels with global contextual and boundary features of transparent objects.
- We conducted exhaustive experiments on five public datasets. Compared with more than 20 different algorithms, our method achieved stable state-of-the-art performance for transparent object segmentation via quantitative evaluation and visual comparison.

## 2. Related Work

### 2.1. CNN-Based Segmentation Methods

CNN-based methods have advanced significantly in semantic segmentation, spawning mainstream CNN backbone models: Ref. [8] proposed a shallow CNN architecture with a novel backbone and fusion convolutional module to optimize glass segmentation; Ref. [9] adopted ResNet as the backbone and designed a context-aware contrast convolution module; Ref. [10] used CNN as the spatial feature extraction backbone and added convolution modules to provide boundary prior information for accuracy improvement; Ref. [11] highlighted the importance of shallow and deep features in capturing fine details and high-level semantics; Ref. [12] and Ji [13] both adopted CNN as the backbone, with the former designing differential modules and edge-aware graph convolution for boundary modeling, and the latter constructing context-aware encoders for glass detection; Ref. [14] enhanced global context modeling via dynamic convolution, while Ref. [15] utilized ResNet for feature extraction and designed specialized convolutional modules with mask supervision; Ref. [16] adopted DeepLabV3 as the baseline and integrated two CNN-based fusion modules with mask guidance; Ref. [3] proposed the lightweight PGSNet and built the matched PGSD panoramic glass dataset to tackle the limitations of existing panoramic glass segmentation methods; Yue et al. [17] proposed ACENet for polyp segmentation to explore adaptive multi-scale spatial context—however, it lacked explicit frequency decomposition and could not decouple high-frequency edges and low-frequency background cues for transparent object segmentation. Pure CNN-based approaches are inherently constrained by localized convolutional receptive fields, resulting in insufficient global context modeling capability. For transparent object segmentation, severe background aliasing and blurred transparent boundaries cannot be effectively distinguished via local convolution operations, which leads to incomplete semantic representation and degraded segmentation robustness.

### 2.2. Transformer-Based Segmentation Methods

In recent years, Transformers have dominated computer vision. By constructing QKV encoders from input sequences, they capture rich contextual cues and robust global semantics: Ref. [18] proposed a Transformer head with a global receptive field to achieve fine-grained segmentation of transparent objects; TGSNet [19] proposed a ViT-based deep architecture to aggregate multi-layer receptive field features and optimize feature fusion performance; Ref. [20] adopted Swin Transformer [21] as the backbone for feature extraction, and utilized blurred region ratios as physical priors to optimize glass segmentation; CAGNet [22] incorporates two distinct convolutional attention mechanisms and integrates them with a Transformer conversion head to implement deep–shallow feature analysis and fusion; Ref. [23] further introduced a Transformer encoder–decoder architecture to enhance the utilization of multi-level contextual information. Single Transformer-based methods easily overlook local fine details. When facing transparent objects with ambiguous edges and spatially inhomogeneous reflective noise, pure Transformer-based methods fail to precisely retain high-frequency boundary cues, yielding coarse contour prediction and insufficient feature discrimination between foreground transparent targets and complex backgrounds.

### 2.3. Hybrid-Net Segmentation Architectures

To achieve comprehensive feature extraction, researchers have designed hybrid networks by combining CNN and Transformer architectures for semantic segmentation: Ref. [24] adopted a Transformer-based structure to construct an attention module, embedding contextual correlations into it to establish spatial and semantic connections between objects; Ref. [25] designed a dual-path feature-aware network that leverages both Swin Transformer and CNN to obtain semantic information from remote sensing images; Ref. [26] designed a dual-branch feature extraction network and proposed a hybrid attention-guided fusion enhancement module to improve the accuracy of salient object detection; Ref. [27] proposed a weakly supervised dual-route Transformer DRTNet trained on bounding box annotations for thyroid nodule segmentation; Ref. [28] proposed a dual Swin–Transformer network with an MCC module for RGB-NIR paired glass detection; Ref. [29] designed an optimized dual-branch Transformer with improved lightweight attention and cross-branch fusion for efficient high-resolution crack segmentation; Ref. [30] proposed DMANet equipped with multi-scale external attention blocks and intra-branch cascade fusion-based dual-branch cascade attention for object segmentation; Yu [31] aimed at the dual-branch contrastive pseudo-supervision framework with cross-consistency constraints, multi-scale attention aggregation and boundary-aware contrastive loss; Ref. [32] proposed a bidirectional cross-prompting adapter to realize dual-branch information interaction and complementary feature fusion. Although it achieves effective cross-branch semantic learning, this method lacks explicit frequency-domain feature modeling and fine boundary optimization, and it relies on extra prompt constraints, making it incapable of adapting to transparent object segmentation. Nevertheless, existing hybrid dual-branch networks cannot well adapt to the unique imaging characteristics of transparent objects. They fail to explicitly model frequency-domain feature differences and neglect high-frequency boundary details severely corrupted by light transmission and reflection interference. Moreover, prevailing methods employ naive feature fusion strategies and lack effective bidirectional cross-branch interaction mechanisms, resulting in mismatched feature distribution and insufficient complementary learning. These inherent limitations lead to coarse feature representation and restricted segmentation performance for transparent objects.

To fully leverage boundary and contextual information without introducing additional auxiliary cues, we propose a frequency-oriented dual-branch mutual learning mechanism for frequency feature integration. This mechanism promotes collaborative feature enhancement and enables joint feature mining for transparent objects.

## 3. Methodology

This section elaborates the learning mechanism and architectural design of the proposed frequency-decoupled interactive neural network tailored for transparent object segmentation. We first introduce the overall dual-branch neural learning framework in Section 3.1, which addresses both the inherent frequency representation bottleneck of self-attention and the unique imaging limitations of transparent targets. Section 3.2 details the interactive information module (IIM) built for bidirectional cross-branch complementary representation learning. Section 3.3 presents the hybrid adaptive calibration module (HACM) to mitigate inter-branch feature distribution mismatch and strengthen discriminative feature learning. Finally, Section 3.4 illustrates the cascaded aggregation head for hierarchical multi-scale feature refinement.

### 3.1. Overall Architecture

Self-attention within Transformer encoders delivers powerful long-range dependencies, yet it carries an inherent low-pass filtering bias during visual representation learning, as theoretically validated in prior frequency-domain neural analysis [33,34]. Self-attention inherently smooths feature maps, prioritizing low-frequency global structural signals while continuously suppressing high-frequency local variations. Given frequency coordinates (u,v), the constraint |F(Att(X))(u,v)|=1 holds at the zero-frequency point (0,0) to retain global low-frequency semantics, whereas |F(Att(X))(u,v)|<1 for all non-zero coordinates weakens high-frequency feature responses.

As Transformer layers stack depth-wise, this low-pass property accumulates and triggers progressive attenuation of high-frequency components. The asymptotic frequency decay characteristic of multi-layer self-attention learning can be mathematically formalized as:

(1) $$\lim_{i\to \infty }\frac{{∥F_{H}\left(\mathrm{Att}{\left(X\right)}^{i}\right)∥}_{2}}{{∥F_{L}\left(\mathrm{Att}{\left(X\right)}^{i}\right)∥}_{2}}=0$$

(2) $$\lim_{i\to \infty }F_{H}\left(\mathrm{Att}\left(X\right)\right)=0,\;\left(u,v\right)\neq \left(0,0\right)$$

From this frequency-domain perspective, the failure of mainstream single-backbone networks on transparent segmentation can be clearly explained. Pure Transformer architectures tend to progressively attenuate high-frequency information as depth increases, which may weaken the distinguishable edge signals of transparent objects and potentially result in blurry contours, foreground–background aliasing, and false segmentation predictions. By contrast, single CNN encoders are constrained by local receptive fields, which limits their ability to model low-frequency global context. As a result, they cannot effectively suppress large-scale reflective noise distributed across the scene, resulting in false-positive regions in complex industrial environments.

**Figure 2:**
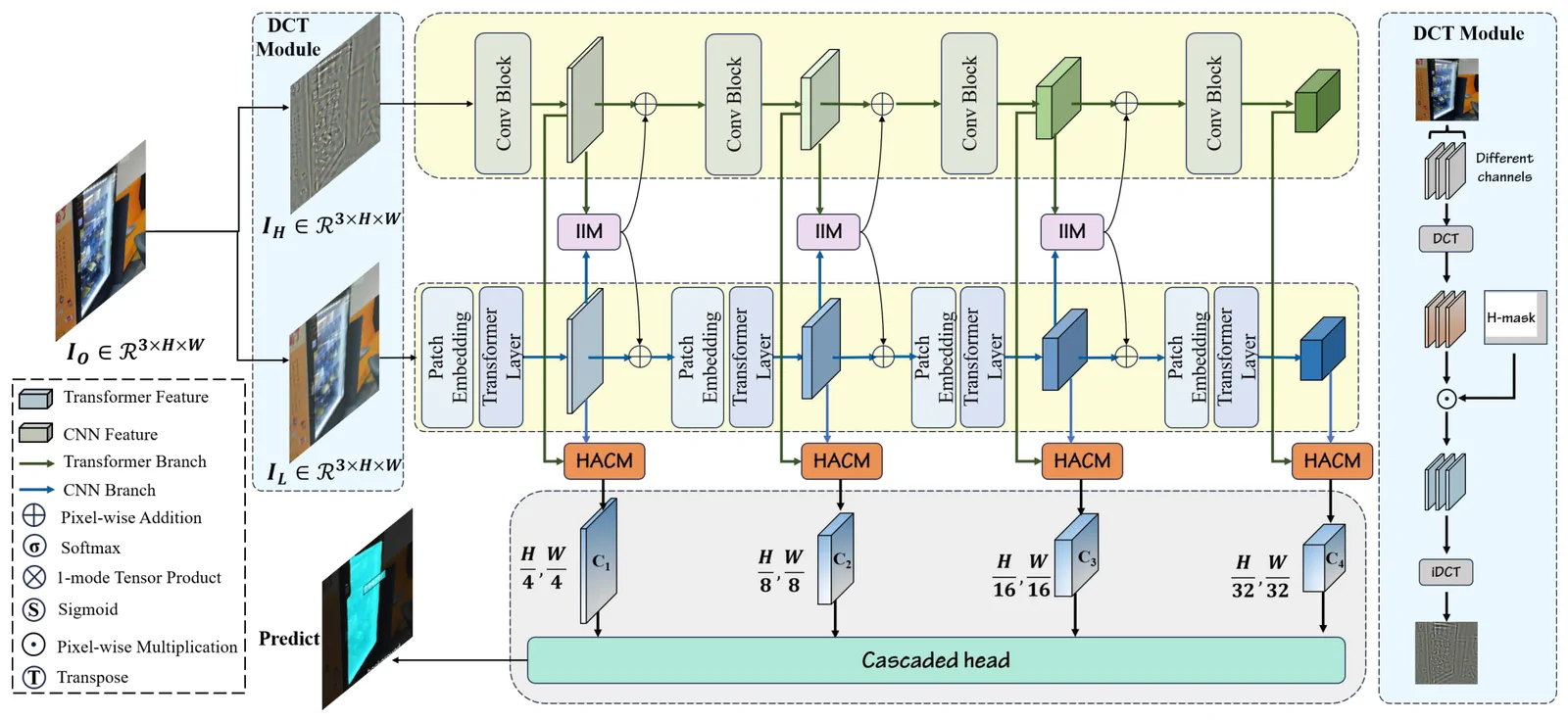
Overview of our proposed model architecture. The model is a two-branch encoder based on P2T and ResNet. The DCT module extracts high- and low-frequency information from the original image. The interactive information module (IIM) interacts and compensates features from two branches. The layer-by-layer hybrid adaptive calibration module (HACM) enhances spatial attention on dual-branch features. The cascade head facilitates cross-layer information.

Frequency-domain decoupling provides a targeted and effective modeling strategy to address this dual challenge. By explicitly separating low-frequency global structural information and high-frequency fine boundary signals before feature encoding, we can assign dedicated learning branches to each frequency component according to their inherent characteristics. The Transformer branch specializes in low-frequency global representation learning, leveraging long-range self-attention to model holistic scene layout and suppress global reflective interference. The parallel CNN branch focuses on extracting high-frequency local edge features, recovering the faint boundary fluctuations unique to transparent objects that vanilla Transformers inevitably discard. Instead of naive stacking of CNN and Transformer modules, we organize our dual-branch encoder into four stages. The dual-branch features at the first three encoder stages are interactively fused and forwarded to their respective next stages. Within the decoder, the dual-branch features of each stage are integrated, and the multi-stage outputs are finally concatenated to produce the prediction map. The entire process is formulated in Figure 2.

Specifically, the 2D discrete cosine transform (DCT) module splits input visual signals into the low-frequency global component $I_{L}$ (3×H×W) and high-frequency boundary component $I_{H}$ (3×H×W). These components are fed into a P2T-small Transformer and a ResNet-50 CNN encoder, respectively. This dual-branch processing produces four stages of multi-scale semantic features $\left\{F_{t}^{1},F_{t}^{2},F_{t}^{3},F_{t}^{4}\right\}$ and boundary features $\left\{F_{c}^{1},F_{c}^{2},F_{c}^{3},F_{c}^{4}\right\}$. Here, DCT is applied channel-wise on the input RGB image *I* (3×H×W) without additional color-space conversion. For each channel, we perform forward DCT to obtain frequency-domain coefficients. A position-dependent binary mask controlled by hyperparameter α is adopted for frequency separation. For the high-frequency component $I_{H}$, hyperparameter α is set to a predefined value: coefficients within the top-left ⌊αH⌋×⌊αW⌋ region are masked to zero to suppress low-frequency content, and the remaining coefficients are preserved. For the low-frequency component $I_{L}$, we retain coefficients inside the top-left ⌊αH⌋×⌊αW⌋ block while zeroing out the remaining high-frequency coefficients. After frequency-domain masking, inverse DCT (iDCT) is performed to map filtered coefficients back to the spatial domain, as shown in the following equation:

(3) $$I_{L},I_{H}=\mathrm{DCT}\left(I\right)$$

where $I_{L}$ (3×H×W) and $I_{H}$ (3×H×W) denote the low-frequency and high-frequency image components, respectively; *I* (3×H×W) represents the input image; DCT denotes the discrete cosine transform module.

To capture cross-branch complementary representations rather than adopting simple feature fusion operations, we devised three dedicated modules. The interactive information module (IIM) performs bidirectional cross-branch projection to mutually enhance global context and transparent object boundary representations, as shown in the following equation:

(4) $$\begin{array}{cc} F_{t}^{1} & ={\mathrm{En}}_{p2t}\left(I_{L}\right) \end{array}$$

(5) $$\begin{array}{cc} F_{c}^{1} & ={\mathrm{En}}_{cnn}\left(I_{H}\right) \end{array}$$

where $En_{p2t}$ and $En_{cnn}$ denote the P2T and ResNet encoders, and $F_{t}^{1}$ and $F_{c}^{1}$ are their outputs at the first stage, respectively. The features from the first three encoder stages are then fed into the IIM to yield interacted features for each branch:

(6) $$F_{tf}^{i},F_{cf}^{i}=\mathrm{IIM}\left(F_{t}^{i},F_{c}^{i}\right),\;i=1,2,3$$

where IIM denotes the interactive information module, and $F_{tf}^{i}$ and $F_{cf}^{i}$ are the outputs at the *i*-th stage. Then, at stages 2 to 4, the features $F_{tf}^{i}$ and $F_{cf}^{i}$ are fed into the encoder blocks of their respective branches. Finally, the feature maps $F_{t}^{i}$ and $F_{c}^{i}$ are obtained for each encoding stage of the dual branches, with spatial dimensions of {H/4×W/4,H/8×W/8,H/16×W/16,H/32×W/32}, respectively. Notably, the feature maps from both branches share identical spatial dimensions:

(7) $$\begin{array}{cc} F_{c}^{i} & ={\mathrm{En}}_{cnn}\left(F_{cf}^{i-1}\right),\;i=2,3,4 \end{array}$$

(8) $$\begin{array}{cc} F_{t}^{i} & ={\mathrm{En}}_{p2t}\left(F_{tf}^{i-1}\right),\;i=2,3,4 \end{array}$$

During the decoding stage, the dual-branch features $F_{t}^{i}$ and $F_{c}^{i}$ from the encoder are fed into the hybrid adaptive calibration module (HACM). These features are fused and dynamically recalibrated to strengthen target discrimination and generalization under occlusion and reflective noise. Through this process, the fused features $F^{i}$ are ultimately obtained:

(9) $$F^{i}=\mathrm{HACM}\left(F_{t}^{i},F_{c}^{i}\right),\;i=1,2,3,4$$

Subsequently, a cascaded multi-scale aggregation head further consolidates the hierarchical semantic-boundary correlations from these fused features and facilitates cross-layer feature propagation for optimized segmentation learning, ultimately yielding the final prediction map $F_{pre}$:

(10) $$F_{pre}=\mathrm{Head}\left(F^{i}\right),\;i=1,2,3,4$$

The full pipeline of our network architecture is illustrated in Figure 2.

### 3.2. Interactive Information Module (IIM)

**Figure 3:**
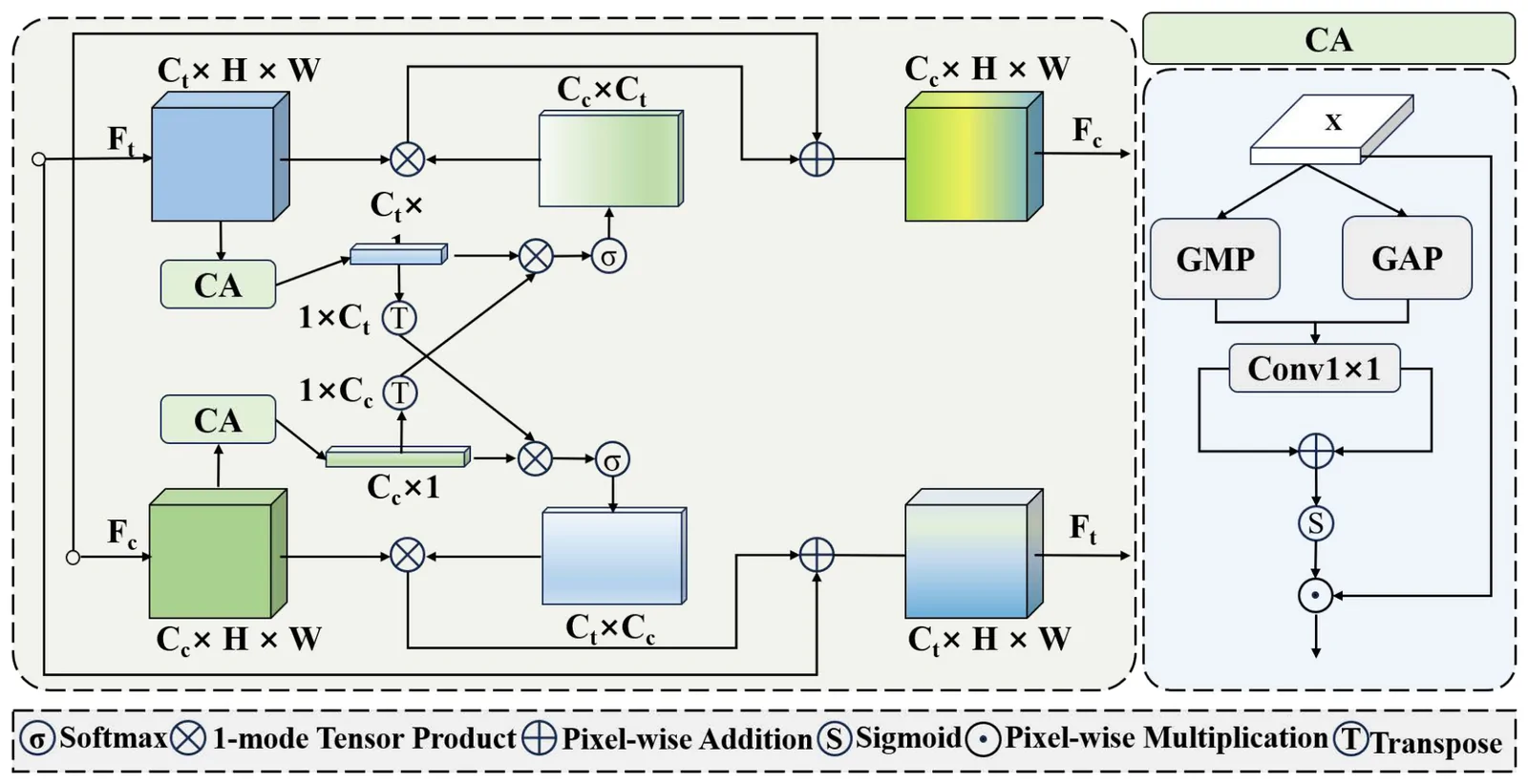
Details of interactive information module (IIM).

This dedicated cross-branch learning unit recovers fine-grained high-frequency boundary representations extracted from the CNN branch, and it embeds such edge cues into the low-frequency global contextual semantics learned by the Transformer encoder, enabling precise localization of transparent object contours guided by holistic scene context, as visualized in Figure 3. To model bidirectional semantic dependencies between the two frequency-decoupled branches, we construct a channel-aware cross-branch correlation matrix. Concretely, the CNN and Transformer encoders output multi-scale feature tensors $F_{c}\in R^{C_{c}\times H\times W}$ and $F_{t}\in R^{C_{t}\times H\times W}$, with $C_{c}$ and $C_{t}$ representing the channel dimensions of each branch. Directly computing full cross-branch channel correlation between raw multi-channel feature maps introduces prohibitive computational overhead and fails to explicitly emphasize semantically dominant channels critical for transparent target discrimination. Since individual feature channels encode disjoint frequency-aware semantics with uneven contribution to segmentation performance, we adopt lightweight Channel Attention (*CA*) to aggregate spatial information and compress each multi-channel feature map into compact channel descriptor vectors ($R^{C_{i}\times H\times W}\to R^{C_{i}\times 1}$). This compression strategy drastically reduces computational complexity while amplifying the responses of task-relevant dominant channels. Following this operation, the original feature tensors $F_{c}$ and $F_{t}$ are condensed into one-dimensional channel vectors $F_{c}\in R^{C_{c}\times 1}$ and $F_{t}\in R^{C_{t}\times 1}$, respectively:

(11) $$F_{\mathrm{ca}}=\mathrm{CA}\left(F_{c}\right),\;F_{\mathrm{ta}}=\mathrm{CA}\left(F_{t}\right)$$

(12) $$\mathrm{CA}\left(x\right)=\sigma_{1}\left(\mathrm{ReLU}\left(A_{G}\left(\mathrm{GAP}\left(x\right)\right)+A_{M}\left(\mathrm{MAP}\left(x\right)\right)\right)\right)$$

where $\sigma_{1}$ denotes the sigmoid function, and $A\in R^{C_{1}\times C_{2}}$ denotes the learnable weight matrix. GAP is the global average pooling operation. MAP represents the maximum pooling operation.

To leverage the complementary strengths of CNN in local detail modeling and Transformer in global context capture, we perform cross-channel interactive feature learning. We construct bidirectional cross-projection channel correlation matrices $Q_{CT}\in R^{C_{t}\times C_{c}}$ and $Q_{TC}\in R^{C_{c}\times C_{t}}$ for dual-branch feature interaction, defined as follows:

(13) $$Q_{CT}=\sigma_{2}\left(F_{\mathrm{ta}}\times F_{\mathrm{ca}}^{T}\right)$$

(14) $$Q_{TC}=\sigma_{2}\left(F_{\mathrm{ca}}\times F_{\mathrm{ta}}^{T}\right)$$

where $\sigma_{2}$ is the softmax function for channel correlation normalization, and T denotes the matrix transpose operation. To enable dual-branch feature maps to capture global context and compensate for the insufficient local details of Transformer features, we fuse features by directly adding projected features $Q_{CT},Q_{TC}$ to original features $F_{t},F_{c}$.

(15) $$F_{tf}=Q_{CT}⊗F_{c}+F_{t}$$

(16) $$F_{cf}=Q_{TC}⊗F_{t}+F_{c}$$

where $F_{tf}\in R^{C_{t}\times H\times W}$ denotes the updated Transformer feature fused with $F_{c}$, and $F_{cf}\in R^{C_{c}\times H\times W}$ denotes the updated CNN feature fused with $F_{t}$; ⊗ denotes the tensor product, which maps the dimensions from $R^{C_{t}\times C_{c}}⊗R^{C_{c}\times H\times W}\to R^{C_{t}\times H\times W}$. These fused features then act as the output of the encoder module and the input to the encoder of the next layer.

### 3.3. Hybrid Adaptive Calibration Module (HACM)

**Figure 4:**
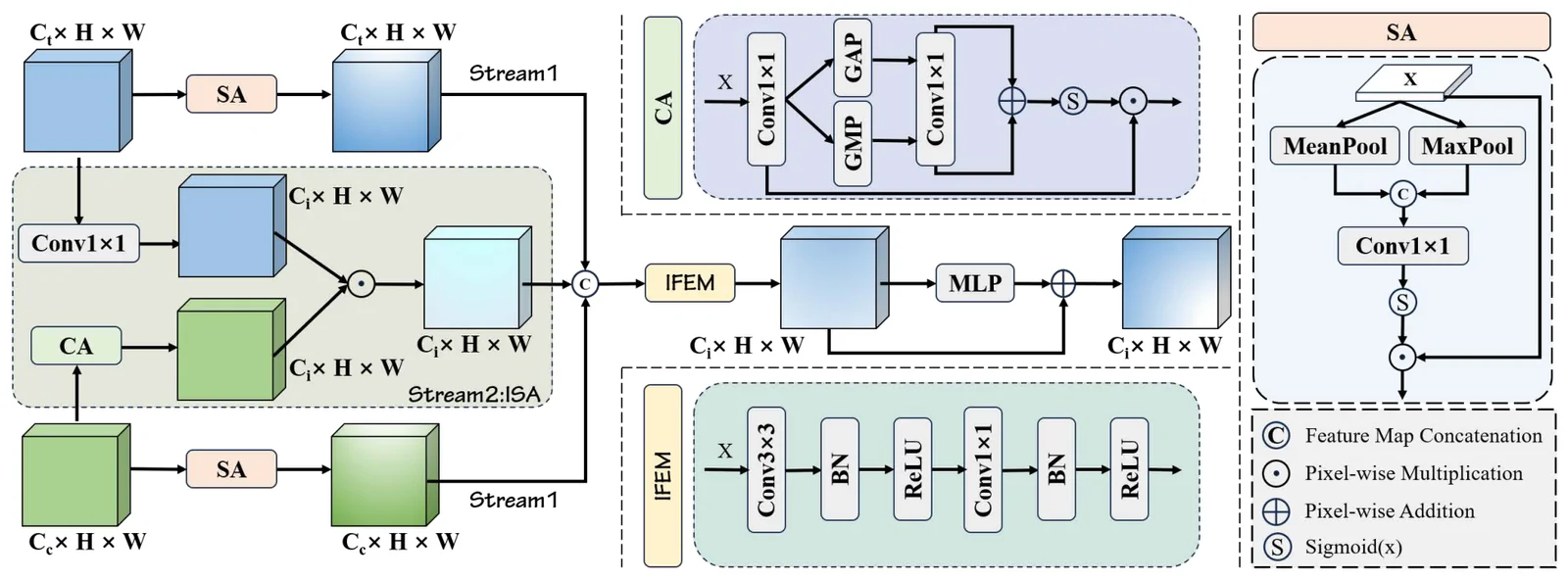
Details of the hybrid adaptive calibration module (HACM).

This subsection introduces the hybrid adaptive calibration module (Figure 4), a tailored neural learning unit for multi-scale cross-branch frequency-complementary representation fusion. It generates unified joint feature $F_{\mathrm{all}}^{i}$ (i=1,2,3,4) via two parallel learning streams. Stream 1 uses spatial self-attention (SSA) to enhance transparent objects’ discriminative high-frequency contours and suppress reflective background noise, with independent spatial attention (SA) refining the features of each branch. Stream 2 adopts interactive spatial attention (ISA) for fine-grained cross-frequency dependency modeling to jointly capture the global low-frequency context and fragile high-frequency boundary cues of transparent targets. We first align feature dimensions for the two branches: the Transformer branch uses 1×1 convolution for matching, while the CNN branch leverages Channel Attention (CA) to eliminate redundant channels and retain valid boundary representations. Dimension-matched features interact via element-wise Hadamard product. The outputs of the two streams are channel-concatenated, refined by a lightweight MLP, and fused with residual skip connections. The mathematical formulation of Stream 2’s ISA unit is given as follows:

(17) $$F_{t}^{i}={\mathrm{Conv}}_{1\times 1}\left(F_{t}\right),\;R^{C_{t}\times H\times W}\to R^{C_{i}\times H\times W}$$

(18) $$F_{c}^{i}=\mathrm{CA}\left(F_{c}\right),\;R^{C_{c}\times H\times W}\to R^{C_{i}\times H\times W}$$

(19) $$F_{in}^{i}={\mathrm{Conv}}_{3\times 3}\left(F_{t}^{i}⊙F_{c}^{i}\right),\;F_{in}^{i}\in R^{C_{i}\times H\times W}$$

where $F_{t}^{i}\in R^{C_{i}\times H\times W}$ denotes the channel-transformed Transformer feature; ⊙ denotes the Hadamard product.

Finally, we conduct further aggregation on the attention-refined features of the dual streams, where IFEM contains a 3×3 convolutional operation, a 1×1 convolutional operation, normalization and ReLU operations:

(20) $$F_{all}=\mathrm{IFEM}\left(\mathrm{Cat}(F_{t}^{\prime },F_{c}^{\prime },F_{in})\right)$$

(21) $$F_{out}=F_{all}+\mathrm{MLP}\left(F_{all}\right)$$

### 3.4. Cascaded Head

**Figure 5:**
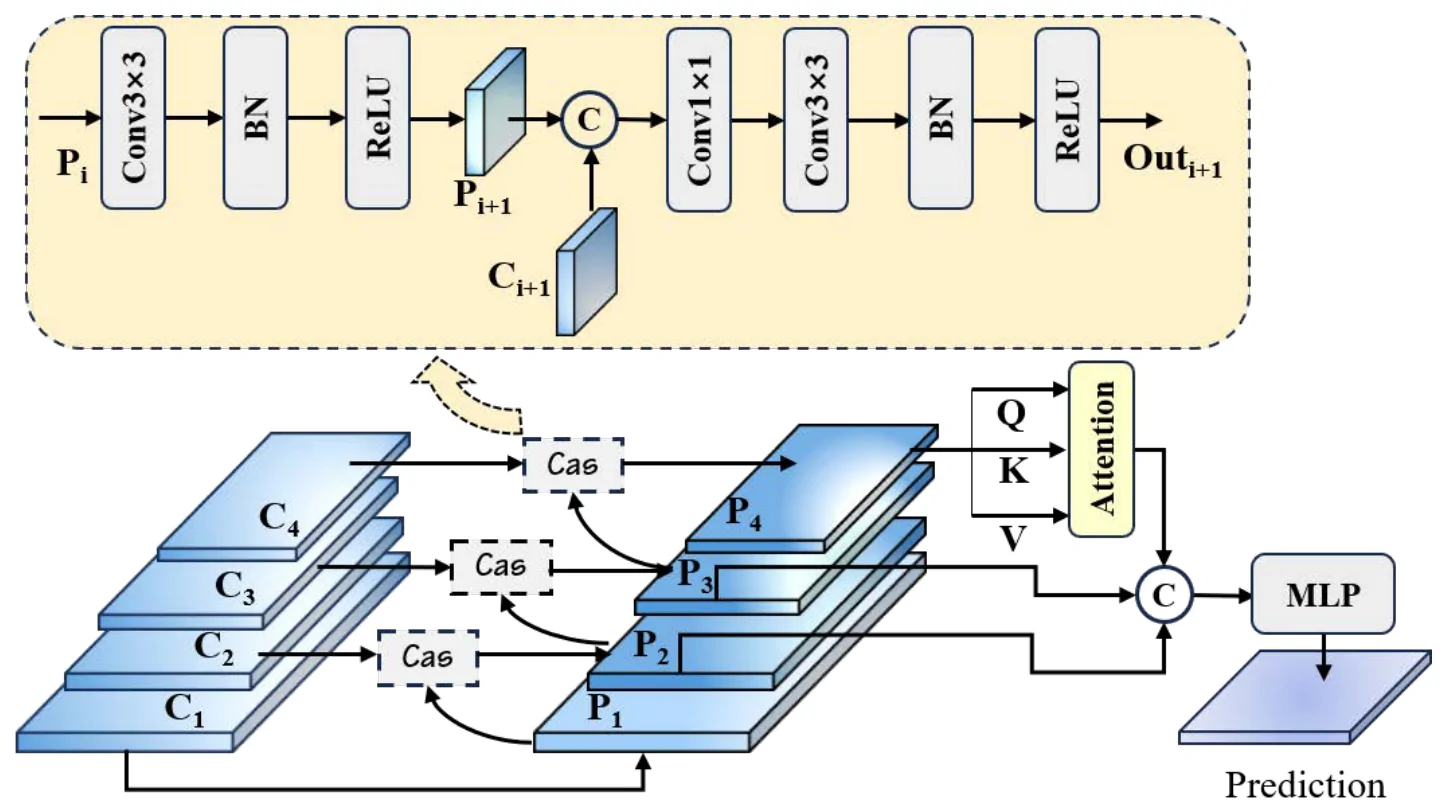
Details of the cascaded head.

For this section, we designed a cascaded inter-layer interaction scheme. Specifically, shallow features encode coarse-grained information, while deep features represent the fine-grained attributes of transparent objects, as shown in (16). By combining coarse-grained information with fine-grained information via convolution blocks, we obtain the comprehensive multi-scale object features in (17). This integration simultaneously captures low-level and high-level features output from HACM across four stages, and it avoids the limitations of the one-sided feature representation in (18). Finally, we aggregate the features from the last three stages and employ the lightweight attention module to further capture the global contexts in (19):

(22) $$F_{p}^{\left(i+1\right)}=\mathrm{ReLU}\left(\mathrm{BN}\left({\mathrm{Conv}}_{3\times 3}\left(F_{p}^{i}\right)\right)\right),\;i=1,2,3$$

(23) $$F_{\mathrm{new}}^{\left(i+1\right)}={\mathrm{Conv}}_{1\times 1}\left(\mathrm{Cat}\left(F_{c}^{\left(i+1\right)},F_{p}^{\left(i+1\right)}\right)\right)$$

(24) $$F_{\mathrm{out}}^{\left(i+1\right)}=\mathrm{ReLU}\left(\mathrm{BN}\left({\mathrm{Conv}}_{3\times 3}\left(F_{\mathrm{new}}^{\left(i+1\right)}\right)\right)\right)$$

(25) $$F_{\mathrm{pre}}=\mathrm{MLP}\left(\mathrm{Cat}\left(F_{\mathrm{out}}^{2},F_{\mathrm{out}}^{3},\mathrm{Attention}\left(F_{\mathrm{out}}^{4}\right)\right)\right)$$

where $F_{p}^{1}=F_{c}^{1}$, $F_{c}^{i}$ denotes the input feature at the *i*-th stage of the decoder (*i* = 0, 1, 2, 3). As the value of *i* increases, the granularity of information becomes progressively finer. $F_{p}^{i}$ denotes the *i*-th stage coarse-grained compensation feature. Attention denotes the multi-head attention module. The overall calculation pipeline is illustrated in Figure 5.

## 4. Experiments and Results

### 4.1. Datasets

Glass Datasets: (1) Trans10k [10] contains two categories (stuff & things) and consists of 5000, 1000 and 4428 images for training, validation and testing, respectively. (2) GDD [9] is a dataset dedicated to glass-like object segmentation, which comprises 2980 training images and 936 test images. (3) Trans10k-v2 [18] annotates 11 fine-grained categories, which are divided into 5000, 1000 and 4428 images for training, validation and testing, respectively.

Mirror Dataset: The MSD [35] dataset contains 3063 images for training and 955 images for testing.

Camouflaged Object Dataset: The MAS [36] dataset contains 1763 training images and 1138 test images.

### 4.2. Evaluation Metrics

Following existing protocols, four metrics were utilized for the performance evaluation: mean intersection over union (mIoU), pixel accuracy (ACC), mean absolute error (MAE) and mean F1-Score. Specifically, mIoU calculated the overlap ratio between predictions and ground truth, ACC reflected pixel-wise classification accuracy, and MAE quantified pixel prediction errors. Referring to prior studies [9], F1-Score, as the harmonic mean of precision and recall, was adopted on MSD and GDD to comprehensively balance detection precision and recall.

### 4.3. Implementation Details

Our implementations were built upon the MMSegmentation toolbox. Unless otherwise noted, all our experiments were run on a single NVIDIA RTX 5090 GPU. The input images were resized to 512×512, and the batch size was set to 4. We employed the AdamW optimizer with an initial learning rate of $6\times 10^{-5}$, betas of (0.9,0.999) and a weight decay of 0.001. Gradient clipping was adopted with a maximum norm of 32. To optimize the training process, we used a parameter-wise weight decay strategy: positional embeddings and class tokens were exempt from weight decay, and the weight decay multiplier for normalization layers was set to 10.0. The learning rate followed a linear warm-up schedule for the first 1500 iterations, after which a poly policy with power 0.9 was applied for up to 160k iterations (320k iterations for the Trans10k-v2 dataset). Cross-entropy loss was used for semantic segmentation. We adopted the default augmentation pipelines from MMSegmentation on all datasets. To ensure a fair comparison, no pre-trained weights were utilized in any experiment. Instead of enforcing identical hyperparameters, which can penalize certain architectures, we followed the recommended settings tailored to each model. Concretely, common segmentation baselines were trained with their official default configurations provided in MMSegmentation, and all competing models strictly adopted the hyperparameters released in their original repositories. Furthermore, all the experiments were conducted with a fixed random seed under deterministic mode, and the best checkpoint was selected according to validation performance.

### 4.4. Comparison Results

We compared our method with state-of-the-art methods from relevant fields to prove the effectiveness of our method, including: salient object detection—U2-Net [37], Spider [38]; CNN-based semantic segmentation—ConvNeXt [39], SegNeXt [40]; Transformer-based semantic segmentation—ViT [41], Swin Transformer [21], P2T [42], MaeNet [43]; hybrid CNN–Transformer semantic segmentation—DSRNet [26], SeMask [44]; transparent object segmentation—Trans2Seg [18], GDNet [9], RFENet [15], GateNet [45].

#### 4.4.1. Salient Transparent Objects Datasets

**Quantitative Comparison:** We quantitatively compared our results with other state-of-the-art methods from relevant fields on the Trans10K, GDD and MSD datasets, and the comparison results in Table 1 testify to the effectiveness of our method. Among all the competing algorithms, our model achieved the best scores on all four metrics across the three datasets. On the Trans10K dataset, our method obtained an mIoU of 90.63%, which surpassed the second-best Segnext-b by 1.17 percentage points, and it reduced the MAE to 0.0401. On the GDD dataset, our mIoU reached 83.21%, with a 1.76-point improvement over Segnext-b. For the MSD dataset, our mIoU was improved by 1.55 percentage points compared with the top-performing P2T-b baseline, accompanied by lower MAE values. Benefiting from the dual-branch architecture, object features were extracted more comprehensively. High-frequency features from the CNN branch accurately supplemented edge details, and co-evolutionary learning between semantic and boundary branches fully exploited their complementary information.

**Figure 6:**
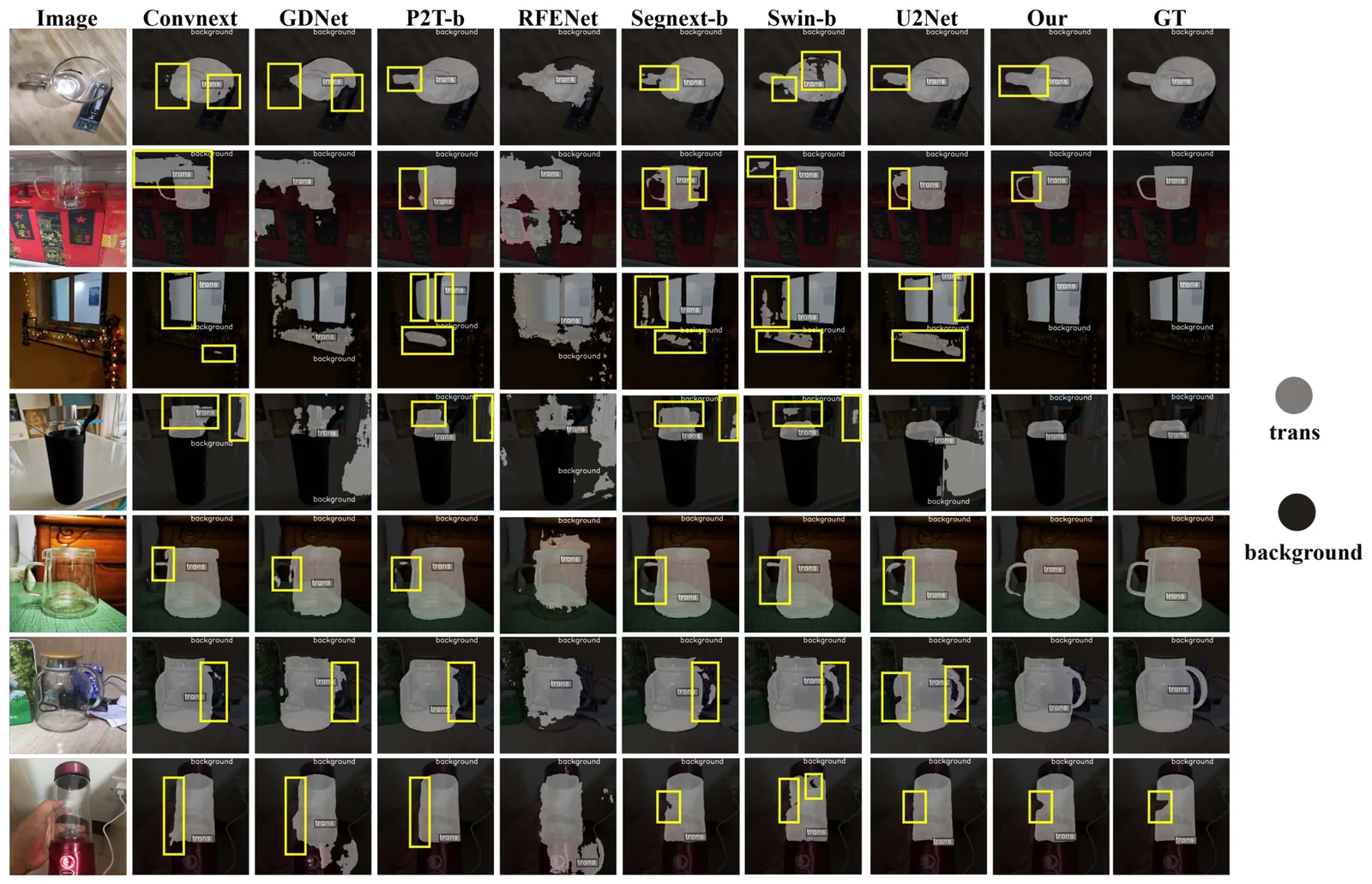
Qualitative comparisons with state-of-the-art methods on Trans10k datasets. The important regions are marked with yellow boxes for emphasis.

**Qualitative Analysis:** To intuitively validate the superiority of our method, we performed qualitative visual comparison against several state-of-the-art approaches on the Trans10k dataset. As shown in Figure 6, key subtle details are marked with yellow bounding boxes for clear observation. Our method preserved richer fine-grained details, achieved accurate target localization while avoiding false recognition in background regions, and it better captured the delicate edge contours of transparent objects. These qualitative results demonstrate that our method can precisely segment transparent objects and extract discriminative semantic features effectively.

#### 4.4.2. Trans10k-v2 Datasets

**Figure 7:**
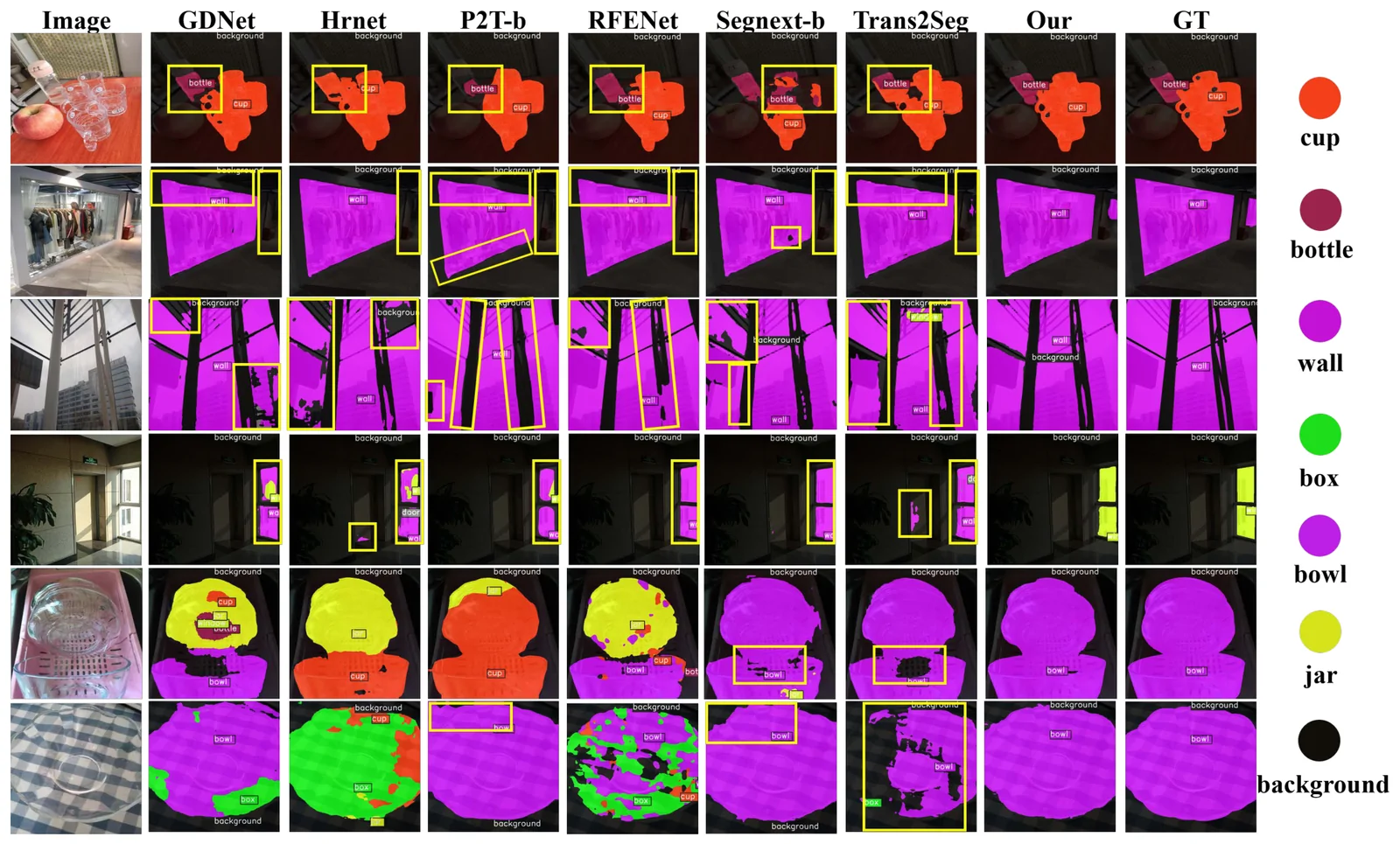
Qualitative comparisons with state-of-the-art methods on Trans10k-v2 datasets. The important regions are marked with yellow boxes for emphasis.

Table 2 reports the specific segmentation performance on Trans10k-v2, with visual comparisons provided in Figure 7. For our method, P2T-small and ResNet-50 served as the encoder backbones of the baseline model, while P2T-base and ResNet-101 were adopted for the Our-large method. Compared with the baseline P2T-small and ResNet-50, the backbone depths were set to (3,4,6,3) and (3,4,23,3), respectively. As shown in the table, our baseline model obtained 65.56% mIoU. Our-large further boosted mIoU to 66.75%. Although RFENet yielded the highest mIoU of 67.10%, Our-large achieved comparable performance and obtained superior aACC and mPrecision scores among all the competitors. Compared with the other methods, our method delivered competitive performance, featuring accurate localization, refined edge representation, and strong robustness against cluttered backgrounds. The quantitative metrics are further supported by the visual examples in Figure 7. These results validate the efficacy of CNN–Transformer frequency-aware fusion for fine-grained transparent object segmentation.

#### 4.4.3. Comparison on FLOPs and Params

**Figure 8:**
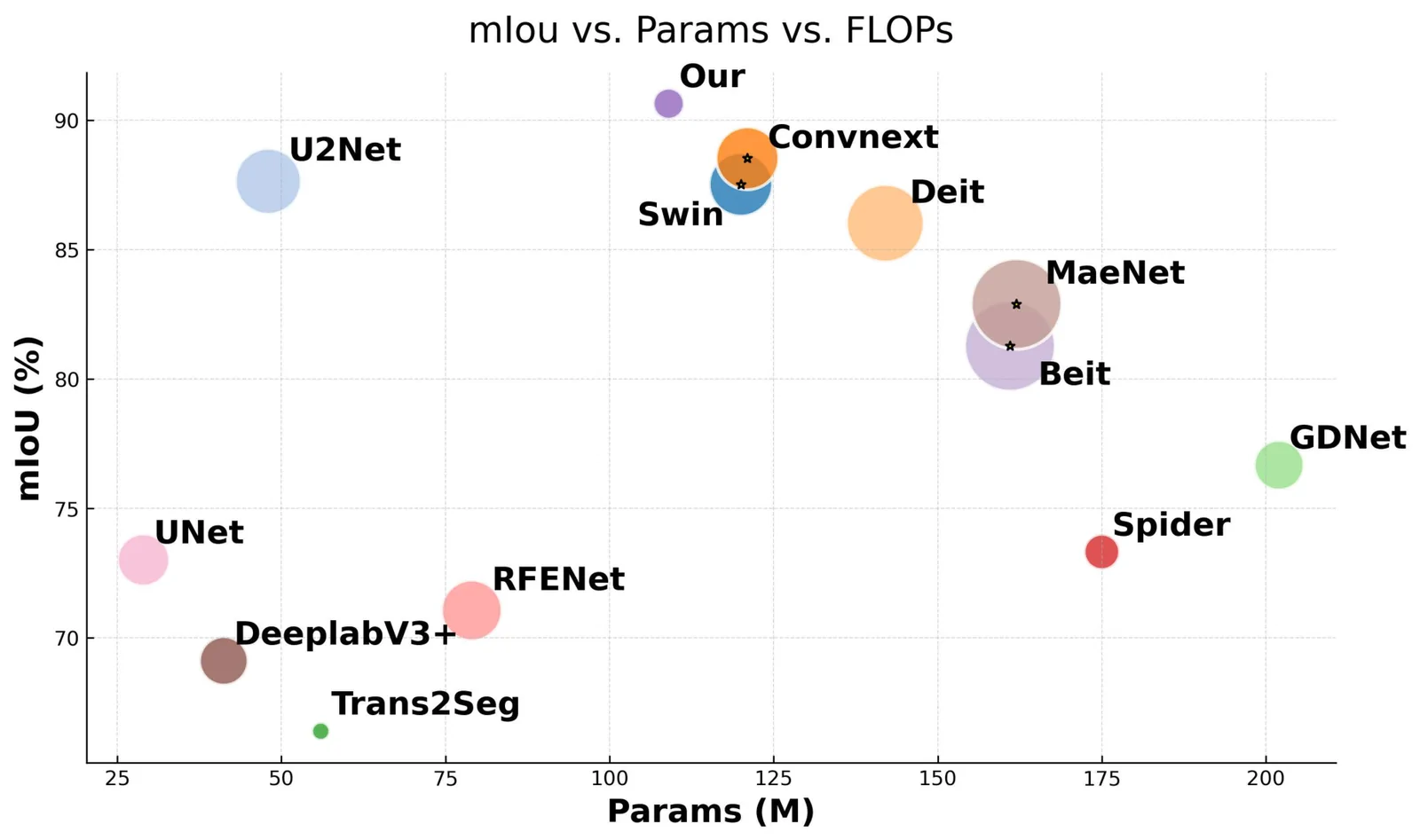
FLOPs and params comparison (The star marker in this figure denotes the center points of the two intersecting circles. It is only used to distinguish the positions of these two circles.).

Furthermore, we compared our method with advanced glass segmentation models in terms of FLOPs and parameters; the numerical values for these metrics are summarized in Table 3. As shown in Figure 8, we took the number of parameters as the horizontal axis, mIoU as the vertical axis, and we used the circle area to represent the FLOPs of each model. Though our dual-branch design brought a modest increase in parameters compared with some of the lightweight baseline methods, our approach achieved competitive segmentation accuracy across all the compared models. In addition, the small circle of our model demonstrates that we kept relatively low FLOPs and maintained a favorable computational cost. Unlike many Transformer-based models that rely on massive parameters and heavy computation to boost performance, our method achieved competitive segmentation accuracy with moderate computational overhead, showing a reasonable trade-off between segmentation performance and computational cost toward real-world deployment scenarios.

#### 4.4.4. Supplementary Experiments on MAS Datasets

**Figure 9:**
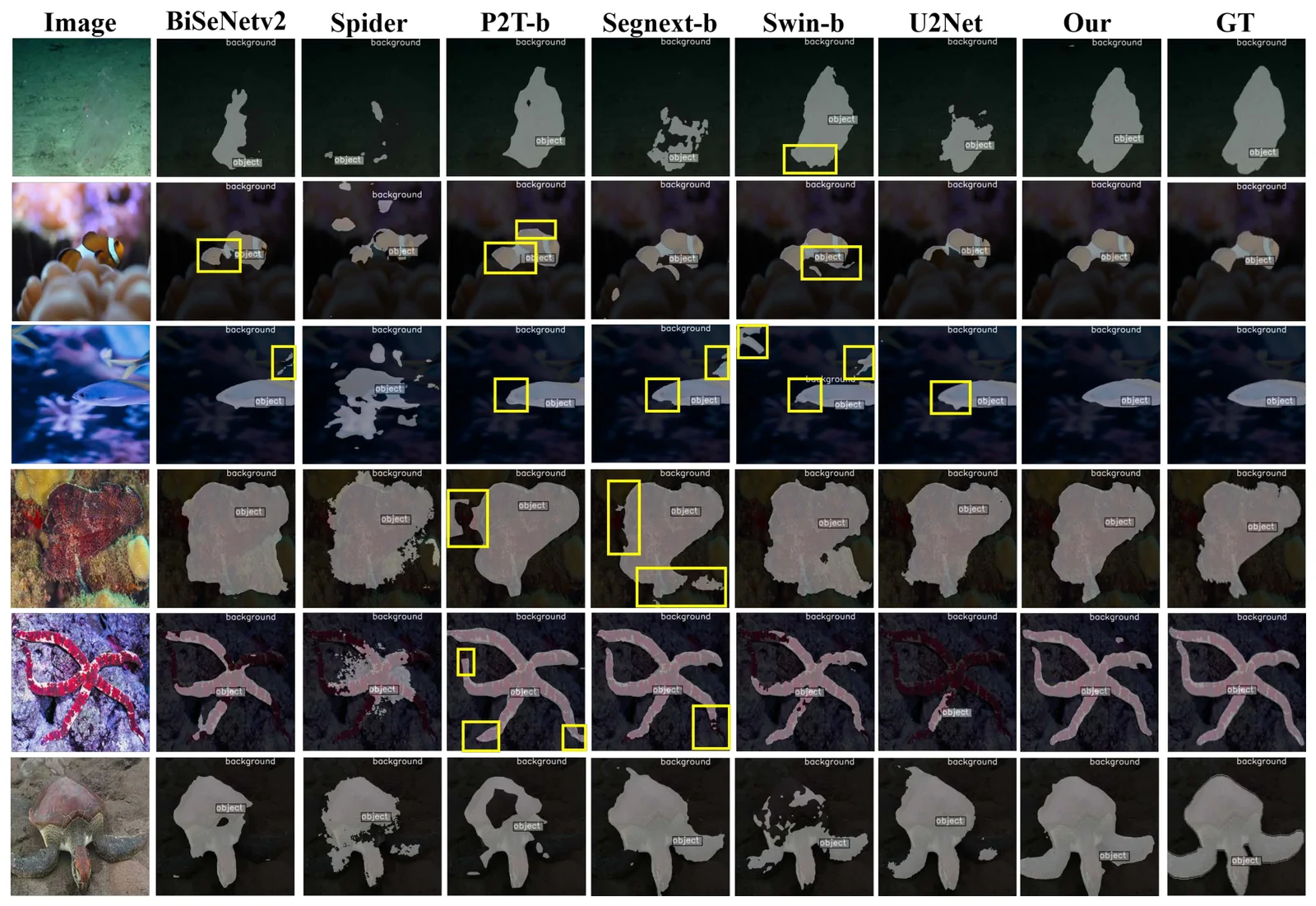
Qualitative comparisons with other methods on MAS datasets. Our method enabled more accurate and complete localization of target regions and generates sharp boundaries. The important regions are marked with yellow boxes for emphasis.

As a supplementary cross-domain test rather than a core benchmark for transparent object segmentation, we conducted tests on the MAS underwater dataset to further verify the generalization ability of our method. This dataset was particularly challenging due to the weak and ambiguous textural features of underwater target objects, complicated background noise and low-light imaging conditions. As quantitatively summarized in Table 4, our method achieved the best performance across all the evaluation metrics, outperforming a series of classic CNN models and advanced Transformer-based segmentation methods. Compared with the second best Segnext-b, our approach obtained a 2.79-point gain in mIoU, raised the F1-Score by 0.0202, and reduced MAE from 0.0641 to 0.0548. Such numerical gains demonstrate our model’s ability to separate foreground objects from texture-similar cluttered underwater backgrounds. The qualitative visualization results in Figure 9 further reveal the advantages of our framework: most of the competing methods showed obvious segmentation errors, such as incomplete foreground regions and blurred edges in the yellow-marked areas, while our model generated more accurate, complete segmentation masks with sharper object boundaries. These observations suggest the promising generalization and robustness of our model toward challenging unseen underwater scenarios.

### 4.5. Ablation Study

#### 4.5.1. Effectiveness of Our Architecture

We conducted ablation studies on three structural settings to validate the rationality of our frequency-guided dual-branch architecture: a single P2T-b backbone, a vanilla ResNet and P2T dual-branch network without frequency extraction, and our complete proposed frequency-aware dual-branch framework. In the vanilla dual-branch baseline, the ResNet branch was designed to capture detailed high-frequency feature information, while the P2T branch was responsible for modeling global contextual features from raw input images. As reported in Table 5, the dual-branch structure achieved obvious performance gains over the single P2T-b backbone on both Trans10k-v2 and MSD datasets, where the mIoU metric rose from 59.95% to 64.93% on Trans10k-v2, which fully demonstrates the superiority of collaborative feature learning via two complementary branches.

**Figure 10:**
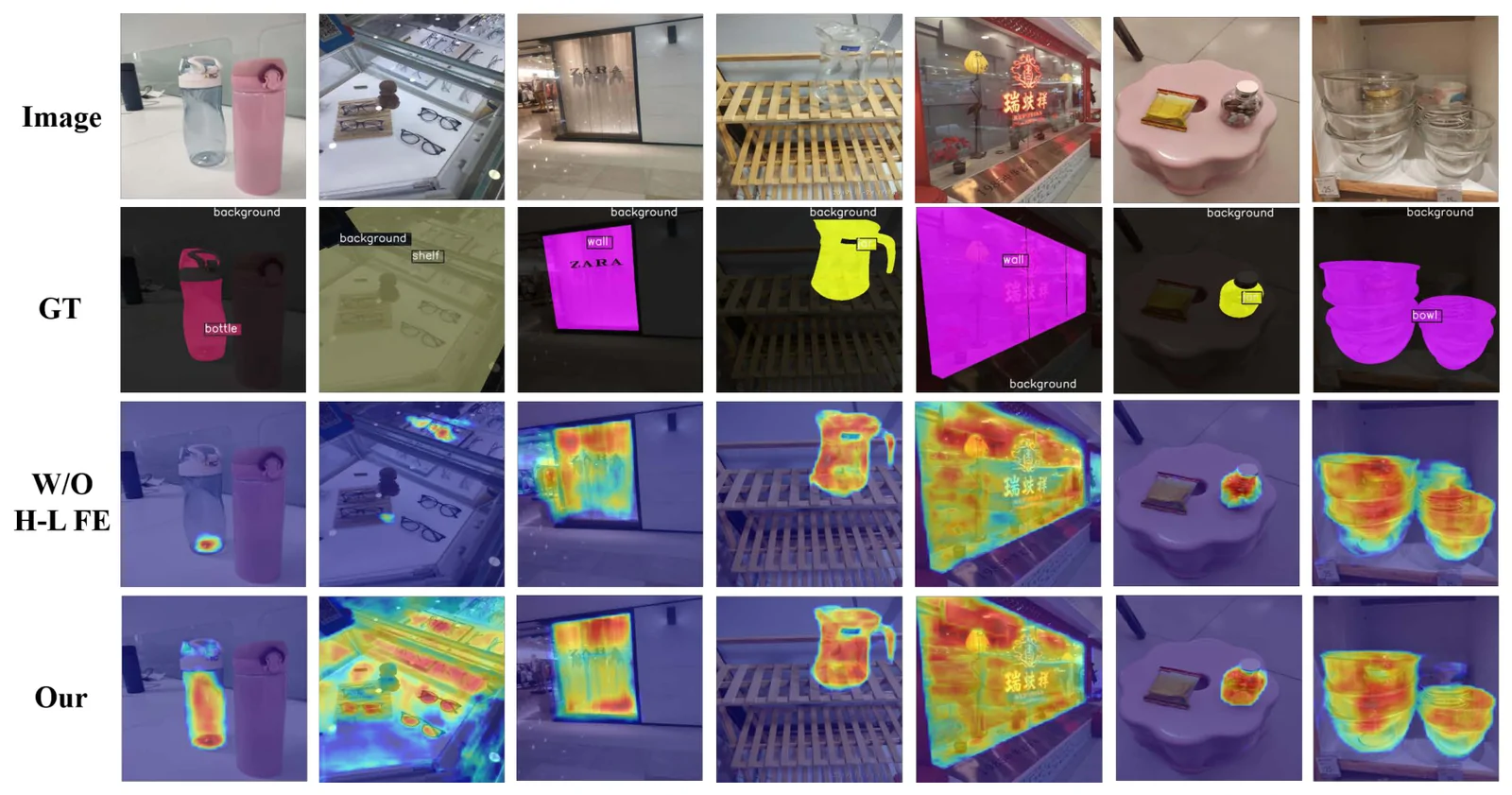
Visualized exhibition for the heat maps of frequency-aware information. The first and second rows show the original images and ground truths, respectively, while the third and fourth rows show the predicted maps without and with frequency extraction, respectively.

**Figure 11:**
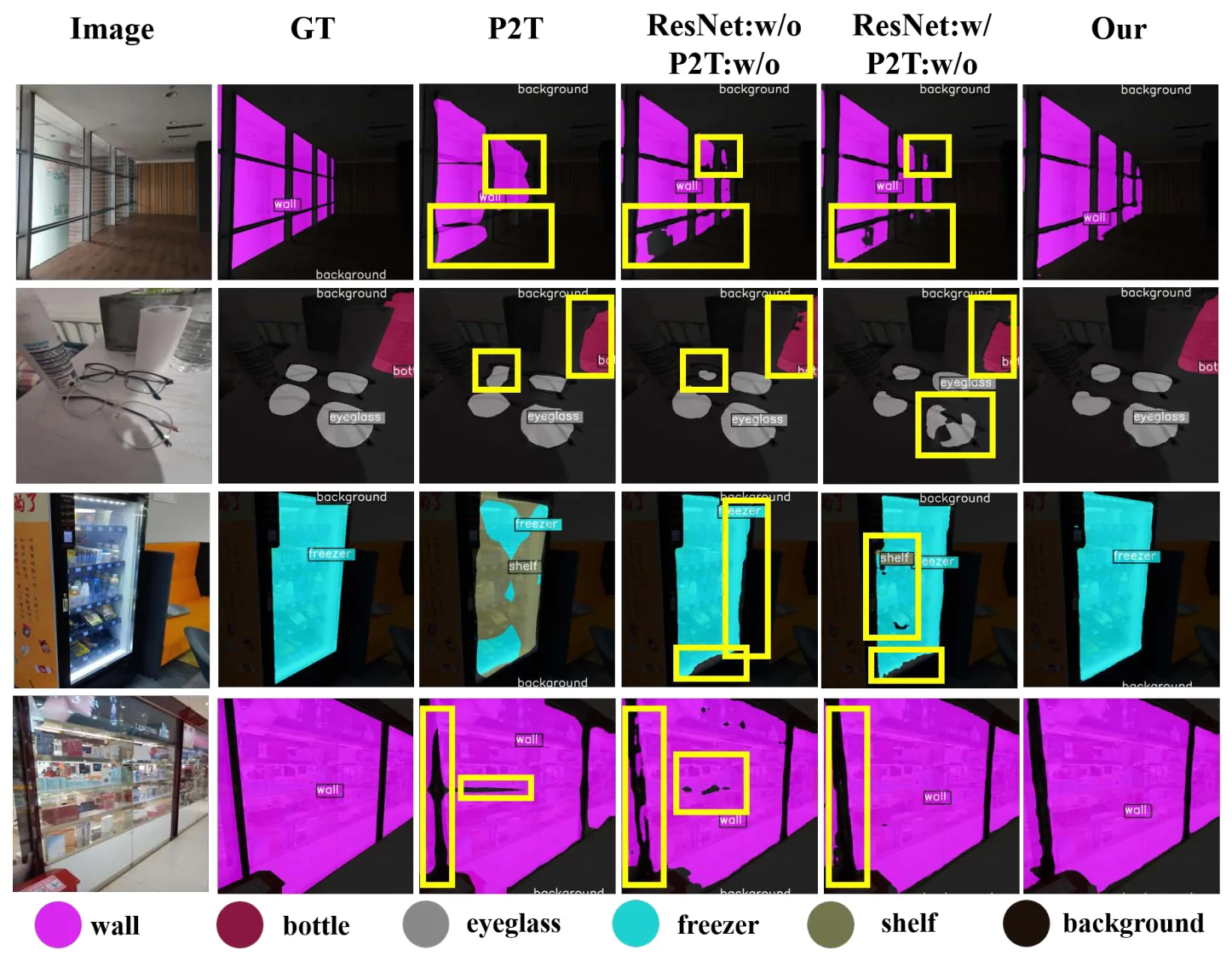
Visualized exhibition for the effectiveness of frequency-aware information and our dual-branch architecture. The first and second columns are original images and their ground truths. The important regions are marked with yellow boxes for emphasis.

Compared with the vanilla dual-branch baseline without frequency extraction, integrating our frequency-aware feature extraction module yielded consistent improvements to mIoU, mFScore, aACC and F1-Score while lowering the MAE metric consistently on both datasets, which quantitatively verifies the effectiveness of our high-frequency CNN branch for fine-grained feature modeling. The feature heatmaps visualized in Figure 10 provide intuitive evidence: the model equipped with high–low frequency extraction could better focus on foreground target regions, activating richer discriminative feature responses on transparent objects and reducing undesired background responses, whereas the variant without frequency guidance only produced sparse and incomplete feature activation. Furthermore, the qualitative segmentation comparisons in Figure 11 reveal that the vanilla dual-branch baseline frequently suffered from missing edges, local under-segmentation and contour distortion within the yellow-boxed critical regions. Benefiting from high–low frequency feature decoupling and cross-branch feature fusion, our full framework generated more complete segmentation masks with smoother, sharper object boundaries for challenging transparent scenes. All the quantitative results in Table 5 and the qualitative visualizations jointly validate that our frequency-guided dual-branch design can effectively fuse local detailed high-frequency features and global contextual low-frequency features to bring steady segmentation performance gains.

In our DCT module, α acted as a control coefficient to balance the contributions of high-frequency and low-frequency features. We swept α from 0.50 to 0.95 on the Trans10k and MSD datasets, and the corresponding results are reported in Table 6. This ablation study aimed to investigate how frequency separation affects transparent object segmentation. Overall, frequency separation yielded performance improvements, yet object discrepancies across different datasets imposed distinct requirements for this hyperparameter. For a fair comparison, we tuned α on the validation set of each dataset while keeping the other hyperparameters fixed. The value that achieved the highest validation mIoU was then chosen as the default for that dataset. The selected value was then kept fixed and applied directly to the corresponding test datasets. The test datasets were used only for final performance evaluation and did not participate in any hyperparameter selection or model selection.

On Trans10k, the model performance remained relatively stable within the investigated range. The highest mIoU and aACC were achieved at an α of 0.95. Performance gradually dropped as α decreased, with the degradation becoming obvious when α fell to 0.50. This was likely because a smaller α introduces excessive high-frequency reflection noise, which interferes with semantic representation. In contrast, the MSD dataset exhibited greater sensitivity to α, where both mIoU and aACC peaked at an α of 0.80. Performance improved with growing α before this point and declined rapidly afterwards. An overly large α tended to suppress high-frequency boundary details, leading to blurred contours and degraded segmentation results. Since Trans10k encompasses a richer variety of transparent object categories, we adopted it as the reference dataset for selecting α. Although MSD achieved its peak accuracy at an α of 0.80, an α of 0.95 still delivered competitive results on MSD. Consequently, we adopted 0.95 as the default α value across all the experiments.

#### 4.5.2. Effectiveness of Interactive Information Module

We ablated the encoder-side interactive interaction module (IIM) on two benchmark datasets by comparing the baseline model without IIM to verify its effectiveness in our dual-branch segmentation framework.

The quantitative results listed in Table 7 reveal consistent performance improvements across all metrics after embedding the IIM. Compared with the baseline model without IIM, our full model achieved steady performance gains on both the Trans10k-v2 and MSD datasets: the mIoU rose from 65.24% to 65.56% on Trans10k-v2, while on the MSD dataset, mIoU, aACC and F1-Score showed measurable improvements and the MAE error was reduced. These results indicate that the IIM module positively facilitated feature collaboration between the two branches, yielding a gain in segmentation accuracy.

**Figure 12:**
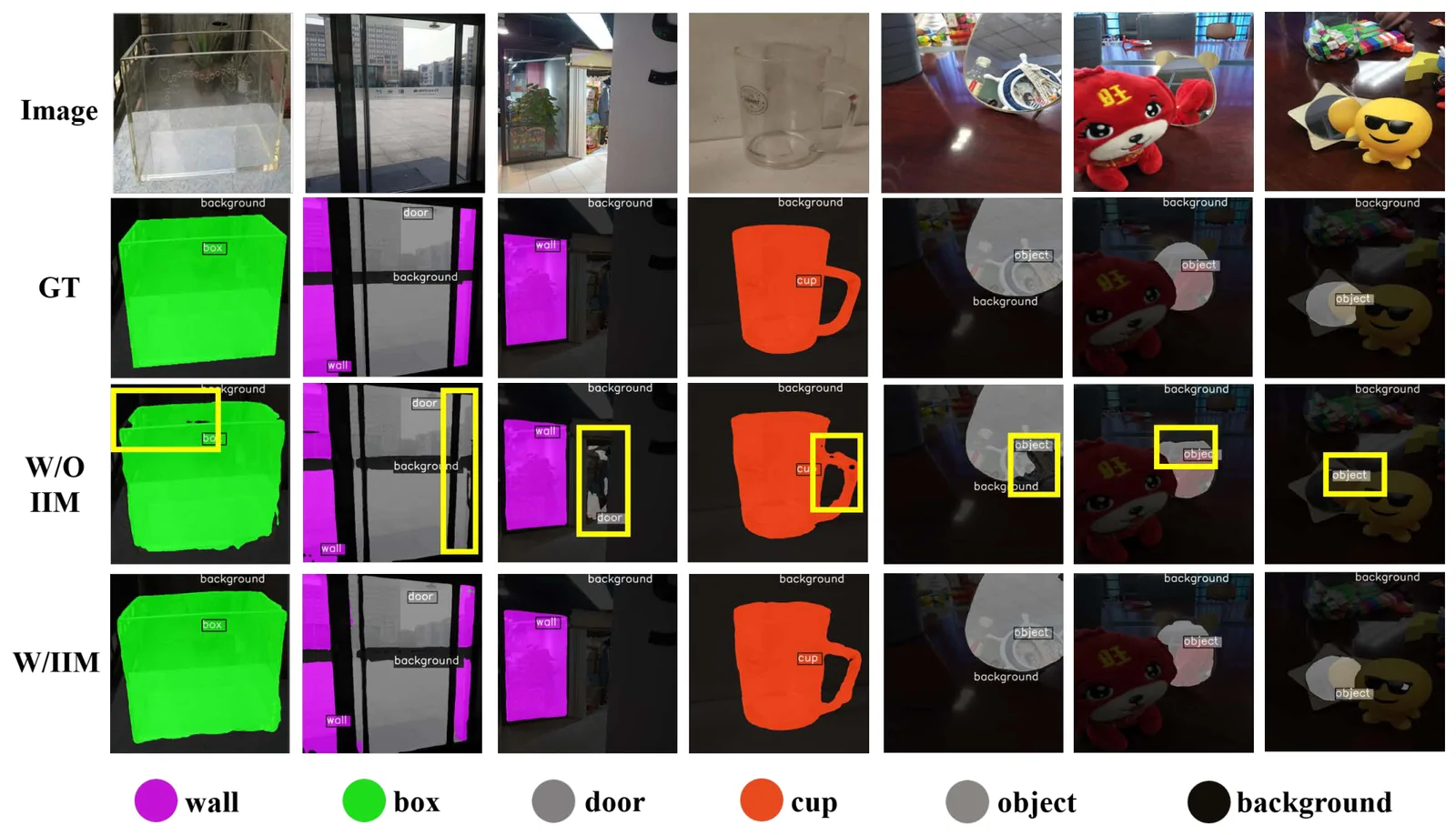
Visualized exhibition for the effectiveness of IIM. The first and second rows are original images and their ground truths, and the third and fourth rows are the predicted maps without or with IIM. The important regions are marked with yellow boxes for emphasis.

As illustrated in the qualitative visual comparisons in Figure 12, the model without IIM showed noticeable boundary omission, incomplete foreground segmentation, and local misclassification within the yellow highlighted regions on various transparent objects. Benefiting from the cross-branch bidirectional feature interaction introduced by IIM, our full model better recovered object contours, generated smoother segmentation boundaries and reduced many local prediction artifacts. These quantitative and qualitative observations suggest that our IIM supports effective cross-branch collaborative feature learning and yields more fine-grained feature representations, which yields measurable performance improvements on challenging transparent object scenes.

#### 4.5.3. Effectiveness of Hybrid Adaptive Calibration Module

We compared the proposed HACM with several representative mainstream attention mechanisms, including PAM [56] and CBAM [57], and we also took the single-stream ISA variant (removing Stream 1 of HACM) for ablation comparison to verify the effectiveness of our dual-stream interactive design.

**Figure 13:**
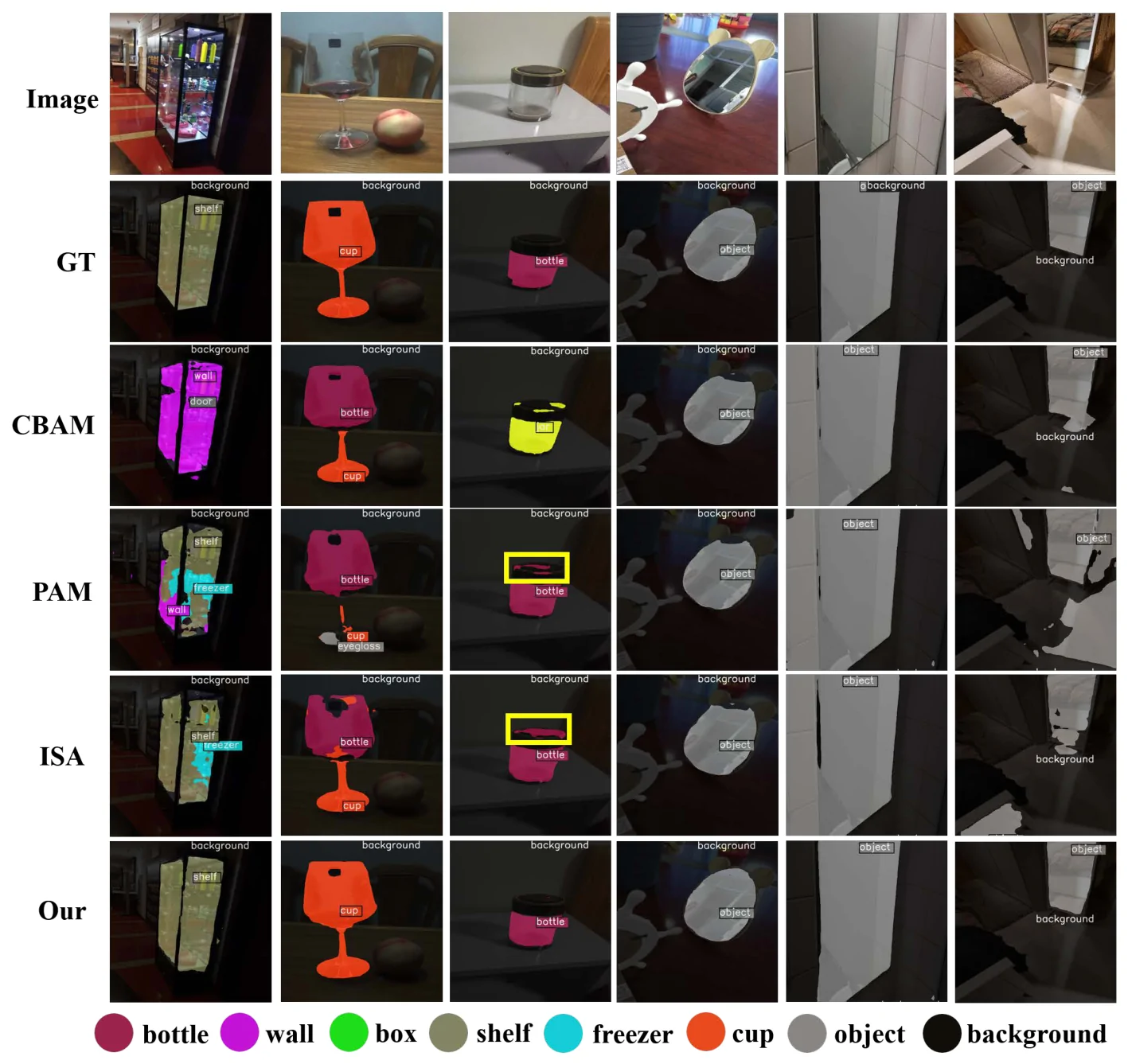
Comparison of different attention methods. From top to bottom: original image, GT, CBAM, PAM, without stream1, and our proposed methods.

The quantitative results reported in Table 8 reveal that all the attention modules yielded slight but positive improvements in segmentation accuracy compared with the baseline. PAM delivered the weakest performance due to its limited feature modeling capacity, while CBAM and single-stream ISA gradually achieved better metric gains by introducing spatial-channel attention and intra-branch context modeling respectively. Our complete HACM achieved the best overall performance on both the Trans10k-v2 and MSD datasets, outperforming PAM, CBAM and ISA on all the evaluation indicators. As displayed in the visual comparison results in Figure 13, PAM and ISA suffered from severe local misclassification and edge missing in typical detail regions highlighted by the yellow boxes, and CBAM still could not perfectly restore the fine boundaries of transparent reflective objects. By contrast, our HACM precisely located all the target regions and generated more complete, smooth segmentation masks by leveraging dual-stream cross-branch feature interaction. These results suggest that our full HACM possesses effective discriminative feature extraction and cross-branch feature interaction capabilities for transparent object segmentation tasks.

#### 4.5.4. Effectiveness of Cascaded Head

To quantitatively verify the contribution of our proposed cascaded head, we conducted ablation experiments on two transparent object segmentation datasets, Trans10k-v2 and MSD. As listed in Table 9, we compared four different settings: removing the cascaded head (w/o), replacing our head with the classic FCN decoder, replacing with the PSP decoder, and our full model equipped with the cascaded head (w). When the cascaded head was discarded (w/o), the model directly adopted the fourth-stage feature map for segmentation. On the Trans10k-v2 dataset, the mIoU dropped to 63.79% and mFScore decreased to 76.81%. On the MSD dataset, it attained degraded performance, including mIoU of 76.34, aACC of 90.24, F1-Score of 0.8607, and MAE of 0.0976. We further substituted our cascaded head with two well-established decoding heads, i.e., FCN and PSP head. Both alternative decoders produced inferior results across nearly all the metrics. For Trans10k-v2, FCN yielded mIoU of 60.19% and an mFScore of 73.90%, while PSP delivered similarly poor performance with mIoU of 60.21% and an mFScore of 73.98%. Similar performance degradation could also be observed on the MSD dataset. These results demonstrate that simple single-stage or other conventional multi-scale decoding schemes cannot fully exploit multi-level feature representations for transparent targets.

In comparison, our complete model with the cascaded head achieved noticeably better performance. On the Trans10k-v2 dataset, it obtained an mIoU of 65.56% and an mFScore of 78.26%, bringing respective gains of 1.77% and 1.45% over the second best. On the MSD dataset, this model delivered mIoU of 76.62%, aACC of 90.40%, and an F1-Score of 0.8627, while lowering MAE to 0.0960. We observed moderate but consistent performance gains against the second-best, as well as the FCN-based and PSP-based decoding alternatives. The performance gain stemmed from the intrinsic design of our cascaded head. Unlike conventional decoding heads that merely concatenate multi-scale features, our cascaded inter-layer interaction scheme iteratively refines coarse-grained compensation features and fuses them with fine-grained decoder features through convolution blocks (Equations (16)–(18)). This iterative fusion mechanism enables comprehensive multi-scale feature aggregation, capturing both the low-level contour boundaries and high-level semantic context of transparent objects. Afterwards, lightweight multi-head attention further aggregates multi-stage outputs to model long-range global dependencies (Equation (19)). Instead of relying on one-sided single-resolution feature representations, the cascaded head progressively refines feature granularity across decoder stages, alleviating the difficulty of segmenting ambiguous transparent regions under complex optical interference.

#### 4.5.5. Effectiveness of All Models

Table 10 shows the ablation results for our four core components, including the DCT module, IIM, HACM, and cascaded head. Each row corresponds to the configuration with one module removed while keeping the others retained. Compared with the full model, removing any of these modules led to fluctuating performance across the Trans10k-v2 and MSD datasets.

We first compared the single-branch P2T-b baseline with our dual-branch architecture. As shown in the first row of Table 10, the single-branch P2T-b yielded mIoU of 59.95% and an mFScore of 73.92% on Trans10k-v2. After enabling the dual-branch design, noticeable performance gains could be observed across most of the metrics, which demonstrates the benefit of complementary feature learning from two branches. We then conducted step-wise ablation to investigate the contribution of DCT, IIM, HACM and cascaded head. When DCT was excluded, we observed a moderate drop on Trans10k-v2, while certain metrics on MSD also degraded. Without IIM, the performance remained at a comparable level on Trans10k-v2, and limited metric changes were seen for the MSD dataset. Disabling HACM resulted in slight performance decay on both benchmarks. Removing the cascaded head yielded a relatively noticeable performance drop on Trans10k-v2. Across the two datasets, the complete model integrating all four modules obtained favorable overall metric values. These results demonstrate that each component contributes to the final segmentation performance, and combining these modules yields consistent overall benefits for transparent object segmentation.

### 4.6. Potential Practical Implications

**Figure 14:**
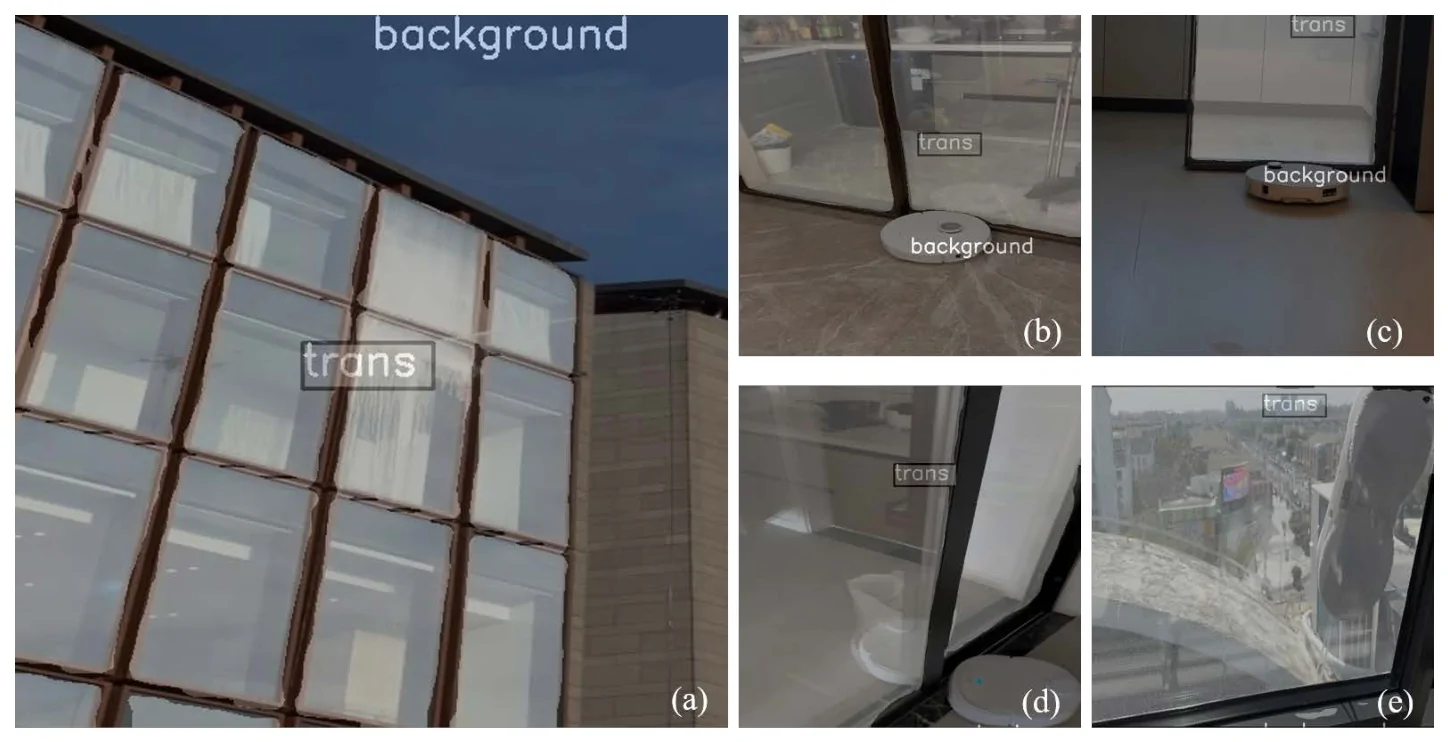
Segmentation performance comparison with other methods. Evaluation results for five cases: (**a**) Glass walls cleaning using UAV. (**b**–**d**) Collision between a cleaning robot and glass doors. (**e**) Glass windows cleaning by wall-cleaning robot.

Transparent targets are ubiquitous within real-world and industrial operating environments. To validate the practical generalization capacity of our proposed neural learning system, we conducted case studies on two representative engineering perception tasks: UAV facility inspection and intelligent robotic visual sensing. As visualized in Figure 14, our frequency-decoupled dual-branch learning network yielded complete, stable and coherent segmentation outputs for industrial transparent infrastructure, including glass doors, transparent partition panels and architectural window glass. Furthermore, to evaluate deployment efficiency, we measured the practical inference speed on the RTX 5090 GPU. Our model achieved a latency of 17.05 ms and a throughput of 58.65 FPS at the resolution of 512×512, which verified its favorable real-time performance. These qualitative results demonstrate that our network has promising potential for industrial transparent-infrastructure inspection tasks, such as UAV inspection and intelligent cleaning-robot perception. Systematic quantitative robotic deployment and closed-loop validation are left for future research. Due to current hardware limitations, we did not have access to robots or UAVs for direct closed-loop deployment tests. Therefore, systematic edge-device profiling and closed-loop robotic validation are left for future work. The proposed learning system shows potential for visual feature perception in automated equipment inspection and autonomous manipulation by intelligent service robots. Additional video qualitative comparisons are provided in the Supplementary Materials.

### 4.7. Failure Cases

**Figure 15:**
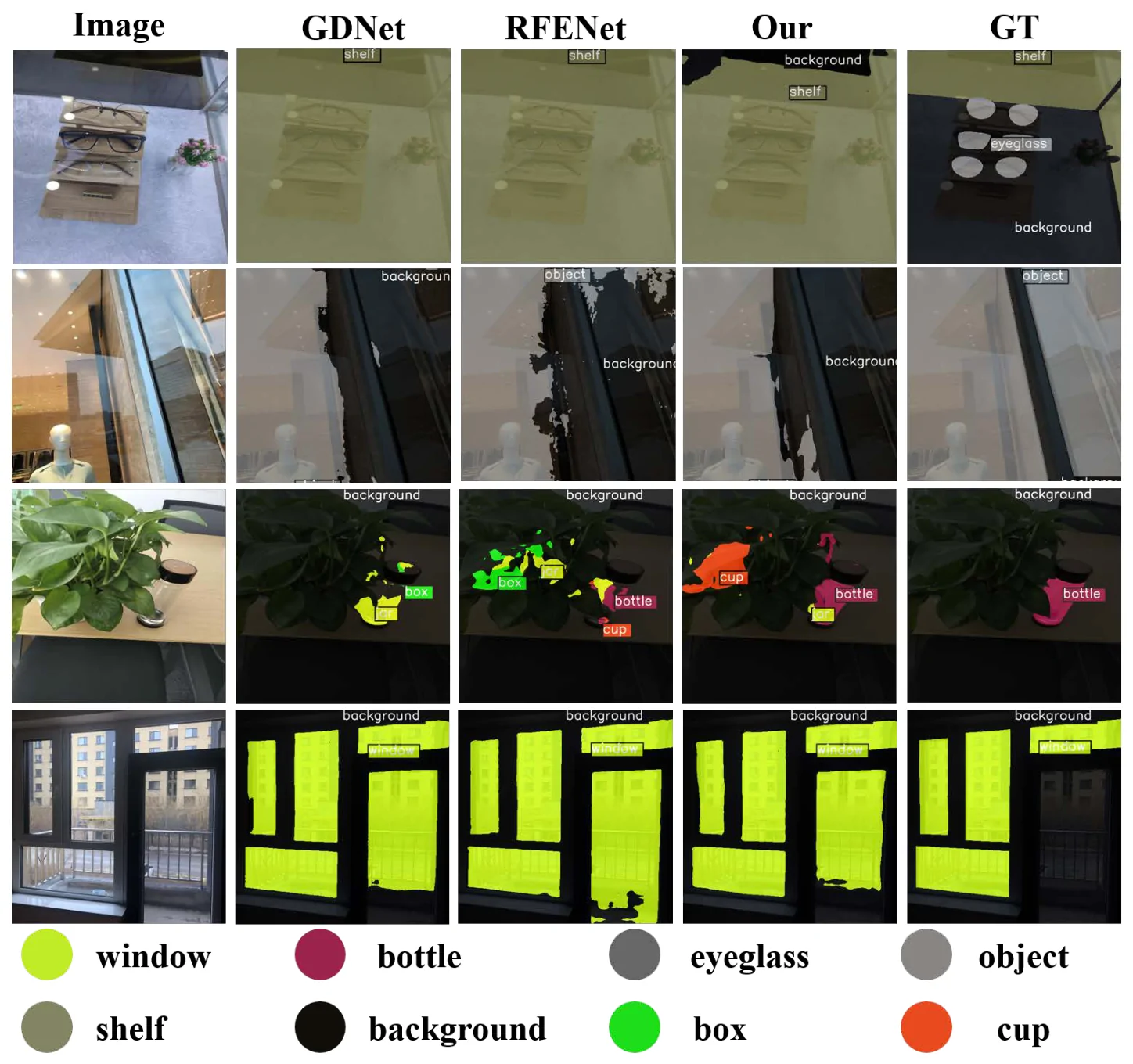
Some failure cases of our method and other methods. The proposed method still encounters difficulties in segmenting transparent objects under challenging conditions, such as similar foreground–background textures, low-light and noisy environments, and extremely small instances.

Some representative failure cases of our method and competing approaches are visualized in Figure 15. Although our frequency-aware dual-branch framework yielded promising performance on most samples, segmentation artifacts still appeared under several tough real-world conditions.

As shown in the first row, when transparent targets exhibited nearly identical texture and intensity patterns to their surrounding background, object boundaries became perceptually indistinguishable. Our CNN branch extracted high-frequency edge cues relying on intensity and texture discontinuities. When foreground–background differences were minimal, valid high-frequency responses vanished. Without reliable edge priors fed into the dual-branch learning pipeline, the Transformer semantic branch could not receive sufficient boundary guidance, eventually generating incomplete masks with missing object regions. The second-row example reflected failure under degraded imaging conditions. Low-light shooting and complex reflection artifacts contaminated raw image signals. Noise and uneven illumination disrupted genuine high-frequency edge components, introducing spurious noisy edges for the CNN branch. These misleading edge features were further propagated to subsequent fusion stages. Since our architecture heavily depends on complementary cooperation between semantic features and edge information, corrupted edge signals break the balance of dual-branch collaboration and produce blurry, discontinuous segmentation contours. For small transparent instances in complex cluttered scenes (third row), the number of effective target pixels was very limited. The down-sampling operations inside both the CNN and Transformer encoders further shrank feature representations for small objects. Sparse feature signals could not provide enough discriminative semantic and boundary evidence. Consequently, both branches struggled to capture compact object outlines, which yielded misclassified regions and fragmentary segmentation outputs. As illustrated in the fourth row, scenes containing multiple adjacent transparent objects brought extra challenges. Different transparent instances shared similar optical properties, and their boundaries were tightly adjacent or mutually occluded. The frequency-guided fusion module struggled to tell apart boundaries belonging to different transparent targets, resulting in poorly separated boundaries and wrong category assignments for nearby transparent targets.

Collectively, these failure cases reveal key limitations of our framework. Our method depends heavily on visible texture jumps to extract object edges, and performance drops when foreground and background show very little visual distinction. The model is also sensitive to image noise and uneven lighting, and it does not work well for small, closely-packed transparent targets.

For future work, we plan to tackle these limitations. One promising direction is to explore boundary cues that do not purely rely on high-frequency texture information from input images. We will also study feature filtering with noise robustness and scale-guided weighting strategies to boost performance on small and densely distributed transparent objects. It should also be noted that all the evaluations in this work were performed on static images. For real-world robotic applications, dynamic conditions such as camera shake, moving targets and motion blur will further complicate transparent object segmentation. Motion blur can erase critical high-frequency edge information, while varying viewpoints continuously alter the reflection and refraction patterns of transparent objects. These effects impose new challenges to our frequency-aware dual-branch design. Exploration on robotic dynamic scenarios remains an open topic for future study.

## 5. Conclusions

Transparent object segmentation is severely troubled by blurred boundaries and poor feature discriminability. These issues stem from the unique transmission and reflection properties of transparent objects, as well as the inherent limitations of robot image sensors, and they greatly hinder reliable visual perception for industrial robots, cleaning robots and UAVs. This paper proposes a frequency collaborative dual-branch network to address the above problems. The network separately processes the high-frequency boundary details and low-frequency semantic information of transparent objects to achieve targeted feature learning. Specifically, a cross-branch IIM is embedded to realize bidirectional feature compensation and fusion between low- and high-frequency features. The decoder integrates an HACM to unify local boundary cues and global contexts, narrowing dual-branch feature gaps for more expressive feature learning. Meanwhile, cascaded inter-layer connections promote sufficient cross-layer information delivery. Our extensive experiments on four transparent object datasets and one camouflaged object dataset, with comparisons against more than 20 state-of-the-art methods, demonstrated that this method effectively compensates for the poor transparent object perception capability of traditional image sensors, and it shows potential as a promising approach for robot visual perception under complex scenarios. However, further quantitative closed-loop validation on physical robotic platforms is still required to fully establish its practical applicability.

## Figures and Tables

**Table 1:** Quantitative comparison results on Trans10k, GDD, and MSD datasets; the three best results are marked in red, blue and green, respectively, the same applies to the other tables.

Methods	Trans10k				GDD				MSD			
	mIoU↑	aACC↑	F1-Score↑	MAE↓	mIoU↑	aACC↑	F1-Score↑	MAE↓	mIoU↑	aACC↑	F1-Score↑	MAE↓
PSPNet [46]	66.20	83.15	0.7893	0.1685	56.95	72.67	0.7255	0.2733	48.84	75.73	0.6176	0.2427
MobilenetV3 [47]	77.19	89.23	0.8685	0.1077	71.04	83.07	0.8307	0.1693	66.21	85.04	0.7851	0.1496
Deeplabv3+ [48]	69.10	84.95	0.8111	0.1505	64.02	78.06	0.7806	0.2194	54.57	79.46	0.6775	0.2054
BiSeNetv2 [49]	87.69	94.58	0.9336	0.0542	76.66	86.79	0.8679	0.1321	73.21	88.69	0.8387	0.1131
EncNet [50]	60.17	79.44	0.7402	0.2056	49.92	66.66	0.6658	0.3334	45.73	72.98	0.5852	0.2702
Convnext-b [39]	88.52	95.01	0.9384	0.0499	80.22	89.03	0.8903	0.1097	72.08	87.83	0.8309	0.1217
Segnext-b [40]	89.46	95.44	0.9438	0.0456	81.45	89.79	0.8978	0.1021	73.24	88.18	0.8397	0.1182
Poolformer [51]	86.91	94.21	0.9291	0.0579	58.05	73.47	0.7345	0.2653	71.34	88.01	0.8245	0.1199
Vit-b [41]	86.01	93.80	0.9238	0.0620	75.81	86.24	0.8624	0.1376	57.52	81.05	0.7068	0.1895
Swin-b [21]	87.51	94.55	0.9326	0.0545	78.80	88.16	0.8814	0.1184	65.60	85.05	0.7794	0.1495
Twins-b [52]	86.98	94.24	0.9295	0.0576	76.93	86.96	0.8696	0.1304	71.80	88.22	0.8279	0.1178
MaeNet-b [43]	82.89	92.31	0.9049	0.0769	74.61	85.47	0.8546	0.1453	56.99	80.81	0.7015	0.1919
P2T-b [42]	88.86	95.16	0.9404	0.0484	80.99	89.51	0.8949	0.1049	75.07	89.79	0.8516	0.1021
Beit-b [53]	81.27	91.53	0.8947	0.0847	72.48	84.06	0.8404	0.1594	56.77	80.25	0.7009	0.1975
Trans2Seg [18]	66.38	83.05	0.7911	0.1695	58.51	73.87	0.7381	0.2613	51.60	75.98	0.6534	0.2402
GDNet [9]	76.66	88.92	0.8650	0.1108	69.89	82.28	0.8228	0.1772	63.48	83.29	0.7633	0.1671
RFENet [15]	71.05	85.25	0.8270	0.1475	66.84	80.13	0.8013	0.1987	63.89	83.96	0.7656	0.1604
GateNet [45]	81.64	91.62	0.8971	0.0838	74.73	85.54	0.8554	0.1446	65.84	90.29	0.7689	0.0971
ICON [54]	69.94	84.46	0.8193	0.1554	68.82	81.55	0.8153	0.1845	61.02	80.65	0.7455	0.1935
SeMask [44]	67.21	83.13	0.7984	0.1687	62.17	76.69	0.7666	0.2331	62.60	87.79	0.7422	0.1221
DSRNet [26]	72.83	86.80	0.8384	0.1320	67.03	80.27	0.8026	0.1973	56.13	85.89	0.6733	0.1411
U2-Net [37]	87.63	94.59	0.9333	0.0541	79.43	88.58	0.8853	0.1142	71.76	87.90	0.8282	0.1210
Spider [38]	73.31	86.97	0.8422	0.1303	66.41	79.84	0.7981	0.2016	61.96	81.57	0.7526	0.1843
Our	**90.63**	**95.99**	**0.9504**	**0.0401**	**83.21**	**90.84**	**0.9083**	**0.0916**	**76.62**	**90.40**	**0.8627**	**0.0960**

**Table 2:** Quantitative comparisons on Trans10k-v2 datasets.

Methods	mIoU↑	aACC↑	mPrecision↑	mRecall↑
MobilenetV3 [47]	47.63	88.44	63.17	63.75
Deeplabv3+ [48]	56.37	91.38	76.46	65.97
BiSeNetv2 [49]	52.79	90.10	70.91	64.76
HRNet [55]	60.43	92.69	82.27	70.30
Convnext-b [39]	61.63	92.05	78.41	73.08
Segnext-b [40]	62.16	92.11	79.92	72.66
Poolformer [51]	39.91	86.30	59.44	54.27
Vit-b [41]	48.85	87.83	69.00	60.97
Swin-b [21]	59.10	91.07	78.99	69.10
Twins-b [52]	54.72	90.36	74.82	66.31
MaeNet-b [43]	47.62	87.51	70.05	58.04
P2T-b [42]	59.95	91.41	76.63	71.90
Beit-b [53]	45.46	86.72	67.06	56.46
Trans2Seg [18]	60.12	91.08	74.99	73.47
GDNet [9]	65.65	93.17	80.49	76.75
RFENet [15]	67.10	93.62	82.05	77.54
Spider [38]	37.11	84.91	57.23	49.98
Our	65.56	92.91	81.17	75.91
Our-large	66.75	93.01	82.72	76.43

**Table 3:** FLOPs and params of some methods.

Metrics	Swin-b [21]	U2-Net [37]	Convnext-b [39]	Vit-b [41]	Trans2Seg [18]	GDNet [9]	Spider [38]	RFENet [15]	Beit-b [53]	Deeplabv3+ [48]	MaeNet-b [43]	UNet	Ours
FLOPs/G	298	322	292	443	27.589	185	96.078	272	604	176	604	203	74.293
Params/M	120	48	121	142	56	202	175	79	161	41.216	162	28.987	109

**Table 4:** Comparisons with other methods on MAS datasets.

Methods	mIoU↑	aACC↑	F1-Score↑	MAE↓
PSPNet [46]	57.96	88.94	0.6848	0.1106
MobilenetV3 [47]	59.88	86.45	0.7164	0.1355
Deeplabv3+ [48]	61.53	89.43	0.7254	0.1057
BiSeNetv2 [49]	73.46	92.88	0.8333	0.0712
EncNet [50]	49.98	86.01	0.5865	0.1399
Convnext-b [39]	72.07	92.33	0.8225	0.0767
Segnext-b [40]	76.01	93.59	0.8531	0.0641
Poolformer [51]	58.70	88.87	0.6942	0.1113
Vit-b [41]	66.14	89.96	0.7727	0.1004
Swin-b [21]	74.02	93.10	0.8377	0.0690
Twins-b [52]	72.27	92.21	0.8245	0.0779
MaeNet-b [43]	67.36	90.78	0.7826	0.0922
P2T-b [42]	74.95	93.22	0.8451	0.0678
Beit-b [53]	67.24	90.75	0.7815	0.0925
Trans2Seg [18]	60.03	89.14	0.7092	0.1086
GDNet [9]	60.54	88.29	0.7179	0.1171
RFENet [15]	55.39	87.27	0.6583	0.1273
GateNet [45]	65.69	84.93	0.7806	0.1507
ICON [54]	64.13	89.99	0.7521	0.1001
SeMask [44]	53.18	78.64	0.6633	0.2136
DSRNet [26]	59.45	81.16	0.7277	0.1884
U2-Net [37]	70.36	92.32	0.8073	0.0768
Spider [38]	63.80	88.69	0.7522	0.1131
Our	**78.80**	**94.52**	**0.8733**	**0.0548**

**Table 5:** Ablation studies of frequency-extraction information and dual-branch architecture.

Frequency Extraction	Backbone	Trans10k-v2		MSD			
		mIoU↑	mFScore↑	mIoU↑	aACC↑	F1-Score↑	MAE↓
w/o	P2T-b	59.95	73.92	75.07	89.79	0.8516	0.1021
w/o	ResNet-50	64.93	77.71	75.42	89.89	0.8542	0.1011
w/o	P2T-s						
w	ResNet-50	65.52	78.20	76.58	90.37	0.8624	0.0963
w/o	P2T-s						
w	ResNet-50	65.56	78.26	76.62	90.40	0.8627	0.0960
w	P2T-s						

**Table 6:** Experiments on the DCT hyperparameter α.

	DCT α	0.95	0.90	0.85	0.80	0.75	0.70	0.65	0.60	0.55	0.50
Trans10k	mIoU↑	90.63	90.38	90.33	90.46	90.22	90.26	90.35	90.39	90.28	89.97
	aACC↑	95.99	95.87	95.85	95.90	95.79	95.81	95.85	95.87	95.81	95.68
MSD	mIoU↑	76.62	75.26	76.70	79.14	78.06	74.83	76.17	76.09	75.58	75.57
	aACC↑	90.40	89.44	90.53	91.64	91.07	89.64	90.12	90.23	90.10	89.76

**Table 7:** Ablation study on interactive interaction module (IIM).

IIM	Trans10k-v2		MSD			
	mIoU↑	mFScore↑	mIoU↑	aACC↑	F1-Score↑	MAE↓
w/o	65.24	77.90	75.84	85.51	0.8572	0.0999
w	65.56	78.26	76.62	90.40	0.8627	0.0960

**Table 8:** Ablation studies of different attention modules.

Method	Backbone	Trans10k-v2		MSD			
		mIoU↑	mFScore↑	mIoU↑	aACC↑	F1-Score↑	MAE↓
PAM	textbfResNet-50	57.90	71.90	71.06	87.59	0.8228	0.1241
	**P2T-s**						
CBAM	**ResNet-50**	65.31	78.00	75.42	89.89	0.8542	0.1011
	textbfP2T-s						
ISA	**ResNet-50**	65.34	78.08	76.58	90.37	0.8624	0.0963
	**P2T-s**						
**HACM**	**ResNet-50**	65.56	78.26	76.62	90.40	0.8627	0.0960
	**P2T-s**						

**Table 9:** Ablation studies of cascaded head.

Method	Trans10k-v2		MSD			
	mIoU↑	mFScore↑	mIoU↑	aACC↑	F1-Score↑	MAE↓
W/O	63.79	76.81	76.34	90.24	0.8607	0.0976
FCN	60.19	73.90	74.07	89.45	0.8443	0.1055
PSP	60.21	73.98	74.38	89.51	0.8466	0.1049
W	65.56	78.26	76.62	90.40	0.8627	0.0960

**Table 10:** Ablation studies of every model.

Dual-Branch	DCT	IIM	HACM	Head	Trans10k-v2		MSD			
					mIoU↑	mFScore↑	mIoU↑	aACC↑	F1-Score↑	MAE↓
P2T-b	W/O	W/O	W/O	W/O	59.95	73.92	75.07	89.79	0.8516	0.1021
W	W/O	W	W	W	64.93	77.71	75.42	89.89	0.8542	0.1011
W	W	W/O	W	W	65.24	77.90	75.84	85.51	0.8572	0.0999
W	W	W	W/O	W	64.96	77.62	74.83	89.36	0.8504	0.1064
W	W	W	W	W/O	63.79	76.81	76.34	90.24	0.8607	0.0976
W	W	W	W	W	65.56	78.26	76.62	90.40	0.8627	0.0960

## Data Availability

The raw datasets analyzed in this study are publicly available from their respective open-access repositories. The complete source code, including the backbone, modules, configuration files, and evaluation scripts, has been made publicly available at https://github.com/LastJazz/Transparent-objects-segmentation (accessed on 29 October 2026) to ensure full reproducibility. Due to the large volume of data, the processed experimental results and visualization outputs can be obtained from the corresponding author upon reasonable request.

## Supplementary Materials

The following supporting information can be downloaded at https://www.mdpi.com/article/10.3390/s26216935/s1, Video S1: Implication video demos.

## Abbreviations

The following abbreviations are used in this manuscript:

CNN	Convolutional Neural Network
IoU	intersection over union
MAE	mean absolute error
mFScore	mean F1-Score
IIM	interactive information module
HACM	hybrid adaptive calibration module
SSA	spatial self-attention
SA	spatial attention
ISA	interactive spatial attention
